# Integrating synthetic accessibility with AI-based generative drug design

**DOI:** 10.1186/s13321-023-00742-8

**Published:** 2023-09-19

**Authors:** Maud Parrot, Hamza Tajmouati, Vinicius Barros Ribeiro da Silva, Brian Ross Atwood, Robin Fourcade, Yann Gaston-Mathé, Nicolas Do Huu, Quentin Perron

**Affiliations:** Iktos, 65 rue de Prony, 75017 Paris, France

**Keywords:** In-silico synthesizability, Retrosynthesis artificial intelligence, machine learning, In silico molecular generation

## Abstract

Generative models are frequently used for de novo design in drug discovery projects to propose new molecules. However, the question of whether or not the generated molecules can be synthesized is not systematically taken into account during generation, even though being able to synthesize the generated molecules is a fundamental requirement for such methods to be useful in practice. Methods have been developed to estimate molecule “synthesizability”, but, so far, there is no consensus on whether or not a molecule is synthesizable. In this paper we introduce the Retro-Score (RScore), which computes a synthetic accessibility score of molecules by performing a full retrosynthetic analysis through our data-driven synthetic planning software Spaya, and its dedicated API: Spaya-API (https://spaya.ai). We start by comparing several synthetic accessibility scores to a binary “chemist score” as estimated by chemists on a bench of generated molecules, as a first experimental validation that the RScore is a reliable synthetic accessibility score. We then describe a pipeline to generate molecules that validate a list of targets while still being easy to synthesize. We further this idea by performing experiments comparing molecular generator outputs across a range of constraints and conditions. We show that the RScore can be learned by a Neural Network, which leads to a new score: RSPred. We demonstrate that using the RScore or RSPred as a constraint during molecular generation enables our molecular generators to produce more synthesizable solutions, with higher diversity. The open-source Python code containing all the scores and the experiments can be found on (https://github.com/iktos/generation-under-synthetic-constraint).

**Graphic Abstract:**

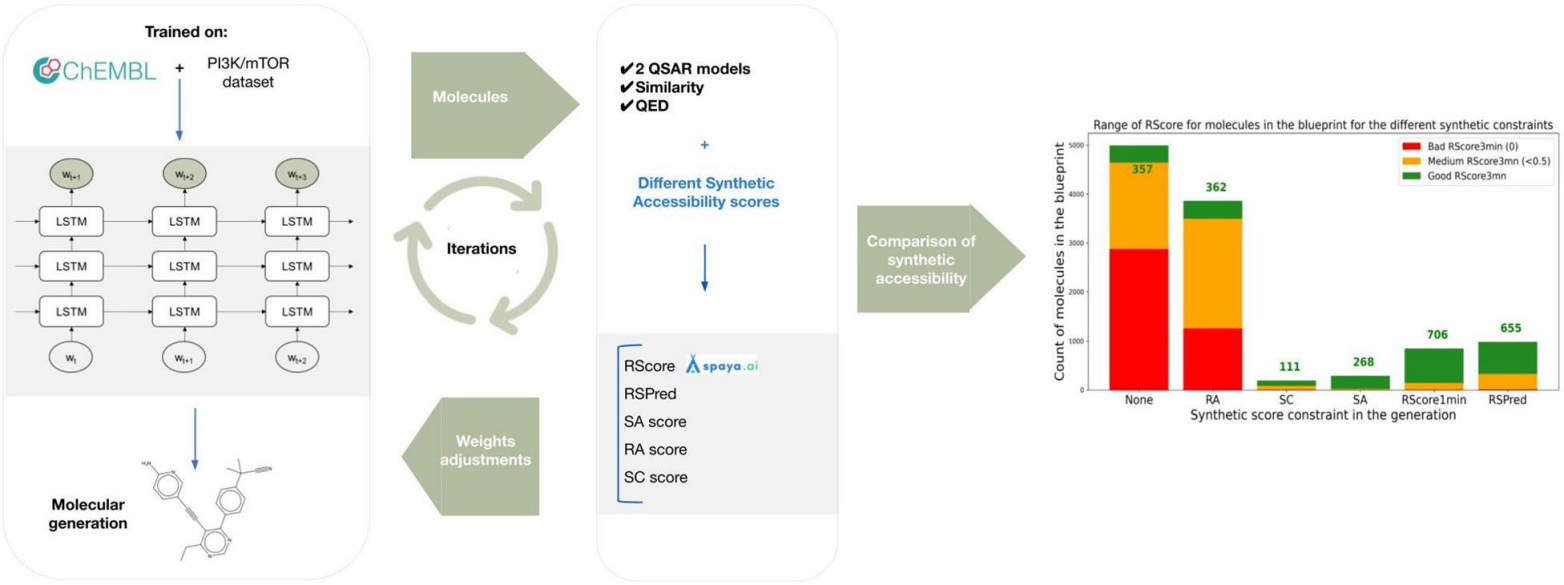

## Introduction

In small molecule drug discovery projects, generative models can be used to design massive libraries of molecules with specific properties [1, 2].

The optimization of an artificial intelligence (AI)-molecular generator to explore a given chemical space and propose new well scored molecules in a Multiparameter Optimization (MPO) project is mostly based on molecular properties and fingerprints [1, 3–7]. However, one of the major challenges in any computer-aided drug design (CADD) project is that the molecules need to be synthesized. Generative models are known to sample many non-accessible molecules [8, 9], and few synthesizability scores are known in the literature to be used in the pipeline of molecular generation [10–13]. Post-processing filters may be applied after the generation to narrow the selection of molecules to those more likely to be synthesizable, for instance AstraZeneca filters [14] include both physiochemical properties and structural filters. No chemical rule is able to completely answer the question of whether a molecule with a valid SMILES can be synthesized or not. Moreover, the evaluation of such scores is challenging, particularly due to the difficulty in interpreting the values. A simple way to define synthesizability is with a binary score denoting “synthesizable” or “not synthesizable”. Although a binary score is useful, it has limits, as it does not allow the prioritization of molecules of the same score. A continuous score provides a way to prioritize similar molecules and produces more signal when used as a reward for a de novo drug design algorithm. With the recent efforts of the community, some continuous scores were recently developed to describe synthetic accessibility [15–18]. Those can be based on chemical substructures, domain expertise, or output of models fitting expert scores. However, as two very similar molecules may have different synthetic routes due to a difference in a single functional group or a single bond change, it may be difficult to find a proxy to a true restrosynthetic analysis. The RA score, for retrosynthetic accessibility score [17], is a predictor of the binary score given by the AiZynthFinder retrosynthesis tool [18]. Its values range from 0 to 1, and, according to the score, the higher the value the more optimistic the algorithm is regarding the synthesis of the molecule. The SC score, for synthetic complexity score [15], ranks the molecules and scores them from 1 to 5. The SC score is based on a neural network trained on a corpus of reactions and relies on the assumption that products are more complex than reactants. Molecules with lower SC scores have a better predicted synthesizability profile. Finally, the SA score, for synthetic accessibility score [16], is a heuristic based score where molecular complexity and fragment contributions are used to evaluate synthetic tractability. Low SA scores indicate less complex molecules and consequently more feasible compounds, the SA score goes from 1 to 10.

To address some of the challenges of synthesizability estimation and to help synthetic, medicinal, and computational chemists in CADD projects and related fields, Iktos has developed Spaya [19], a template-based retrosynthesis AI software that computes synthetic routes and ranks them based on a synthesizability score. In this paper, we describe the Retro-Score (RScore), a synthetic accessibility score derived from the output of a full Spaya retrosynthetic analysis for a given molecule, and we compare it with three other synthesizability scores known in the literature (RA score, SC score and SA score). We highlight the importance of conducting a full retrosynthetic analysis to determine synthesizability. The RScore can be used:

1. To evaluate the synthesizability of molecules given by generative models,
2. Inside the generation itself, to guide the generator to an area of the chemical space where molecules are synthesizable.

Because of the computational costs associated with the computing of a full retrosynthetic analysis needed to obtain the RScore, we also describe a new, easier to compute score called RSPred. RSPred is obtained by training a Neural Network on the output of the Spaya RScore and performs similarly well to the RScore in a variety of tasks, but can be computed orders of magnitude faster.

## Methods

### Datasets

The ChEMBL 24 [20] dataset was used, with the same post-processing as described in the Guacamol Benchmark experiments [9]. The post-processed ChEMBL dataset can be downloaded from the following link [21].

Another dataset, that we have named ’Pi3K/mTOR’ [22–24], was also used. It is a library of 463 structurally homogeneous molecules containing values of IC50 for the two targets Pi3K (pKi measured on the Phosphoinositide 3-Kinase) and mTOR (pKi measured on the mechanistic Target Of Rapamycin), from the Chembl database. After the definition of a threshold of activity, pIC50 Pi3K $\ge 7$ and pIC50 mTOR $\ge 8.5$, the molecules active for both targets were removed. The dataset is accessible in the GitHub project associated with this paper [25].

### The RScore from Spaya API

The score of a retrosynthesis route in Spaya is a proprietary score composed of four separate scores as follows,

1 $$\begin{aligned} score\,(route)= f(d,p,c,a) \end{aligned}$$

where:

- d = number of reaction steps in the route
- p = likelihood of the disconnections of the retrosynthesis route predicted by a single step retrosynthesis model
- c = convergence of the route
- a = applicability domain estimation of the reaction templates used to make the disconnections

To simplify the use of the algorithm on large batches of molecules, Iktos has recently launched Spaya-API [19], an API running on Spaya’s algorithmic engine for library scoring purposes, which has been used herein to evaluate the synthetic accessibility of newly generated molecules. For a given molecule (m), the RScore is derived from routes proposed by Spaya, but handled in a high throughput manner by Spaya-API. The lowest RScore value is 0, indicating no route was found by Spaya within a given period of time; and the highest score is 1, where the route is a one-step retrosynthesis exactly matching a reaction described in the literature. To score a molecule and obtain its RScore value, Spaya-API performs a retrosynthetic analysis with an early stopping process. The early stopping mode stops the Spaya run when a route with a score above the predefined threshold (set to 0.6 by default) is found, or after the defined timeout (set to 1 min by default) has elapsed. The RScore of a molecule is defined as:

2 $$\begin{aligned} RScore(m)= \max _{\begin{array}{c} \textit{routes given by Spaya}\\ \textit{with early stopping} \end{array}} \Big ( score\,(route(m)) \Big ) \end{aligned}$$

The score is rounded to one decimal, and hence can take 11 different values (from 0.0 to 1.0). Spaya-API also returns the number of steps for the best synthetic route found for each input molecule. The list of commercial compounds used for the retrosynthesis is a catalog of  60 M commercially available starting materials coming from 17 different providers, the exhaustive list of providers can be found in Additional file 2: Fig. S1. To speed up computation, a default timeout of one minute was set when the RScore was used as a synthetic constraint in generative design experiments (RScore1min). In order to better approximate the output that would be obtained from a comprehensive retrosynthetic search, this timeout was increased to three minutes when the RScore was used for scoring molecules in post-processing (RScore3min). We studied the impact of the timeout on the RScore of 1000 molecules sampled from ChEMBL24 (see Additional files 1, 2). In average, the difference between the RScore1min and the RScore3min is of 0.3, and increasing the timeout beyond 3 min doesn’t increase significantly the RScore value. The complete study can be found in the Additional file 2 (Fig. S2), as well more details about the retrosynthesis technology implemented in Spaya (Fig. S1).

The RScore1min was compared with three synthetic scores previously published in the literature: the RA score [17], the SC score [15], and the SA score [16]. The three packages to compute those scores are available on GitHub [26–28]. These scores were computed on a sample of 5000 molecules from the pre-processed ChEMBL dataset, and were compared with the RScore1min in the section *Comparison of synthetic scores*.

### Prediction of RScore

The RScore1min computation implies a full retrosynthesis, which is time consuming, with an average of 42 s per molecule to trigger the early stopping. For that reason a regression model was built, with the goal of replacing the computation of the RScore1min with a simple neural network inference.

The dataset used was composed of 70K molecules from the pre-processed ChEMBL dataset, and 300K molecules sampled from the generator Guacamol pre-trained on ChEMBL. The molecules were represented by real vectors of the ECFP2 fingerprints with a radius of 2, modulo-folded to size 8192 and then ln(x + 1)-pre-processed. The dataset was split into a training set (90%), a validation set (5%), and a test set (5%).

To build this continuous predictor of the RScore1min, a neural network was trained on features of the molecules. Different values were tested for the neural network configuration and training parameters, the selected parameters were those leading to the best R2 score value between the RScore1min and RSPred on the validation set. The parameter ranges were: number of hidden layers (1, 2, 3, 4), hidden layer size (30, 50, 100, 200), batch normalization (with, without), and dropout (0, 0.01, 0.05, 0.1, 0.2). The model was a feed-forward neural network composed of three hidden layers of size 100, with Relu activation function. After each layer, a batch normalization layer was added [29]. A sigmoid was added as the last activation function. For the training part, a dropout [30] with a probability of 0.05 was used, the loss was the mean squared error, the optimizer was the Adam optimizer [31] with an initiate learning rate of $5\textrm{e}{-5}$, the batch size was 2048. The model was trained until the validation score stops improving for three consecutive epochs, after epoch 6.

### Generations of molecules

For all the generations, the package Guacamol [32] provided by BenevolentAI was used. The generator is a Recurrent Neural Network, containing three layers of Long Short-Term Memory (LSTM) of size 1024. The network was initialized with the weights given by Guacamol on their GitHub project [32], which was obtained by training on the large dataset ChEMBL 24 [20]. For each generation, the reward used was a geometric mean of the different scoring functions on which modifier functions (described in *Score modifiers* section) were applied. The generators were optimized in order to sample molecules that have a good reward. The optimization algorithm used was the Hill Climbing MLE (Maximum Likelihood Estimation) [33] [1], in which at each step 1024 molecules are being sampled from the generator, then scored, and 2 epochs of teacher forcing [34] are performed on the top scored 152 molecules. Overall 49 generations were run: 3 generations for in-silico validation of the RScore (presented in the next section), 40 Guacamol generations (one generation without synthetic constraint and three with synthetic constraint for each of the 10 tasks), and 6 Pi3K/mTOR generations (one generation without synthetic constraint and five with synthetic constraint). The implementations of those generations can be found on GitHub [25].

#### In-silico validation of the RScore

In this section the selected synthetic scores of the literature are evaluated regarding their quality as proxies of synthetic accessibility. This experiment serves as a first justification for using RScore3min as ground truth of synthetic accessibility in the other experiments described in the paper.

The experiment consists in:

1. Sampling molecules from 3 different similarity constrained generation runs
2. Selecting a bench of molecules from those samples for synthetic accessibility assessment
3. Asking chemists to label each molecule as either feasible or not feasible
4. Assessing how well the different scores discriminate feasible and non feasible molecules as assessed by chemists

The generator used was exactly the one presented in the previous section, and the reward was Tanimoto Similarity on ECFP4 fingerprints (computed with a radius of 2 and 8192 bits) to a target molecule. The number of hill climbing steps was 300. The three reference molecules selected were imatinib, acetylsalicylic acid and a molecule from the Pi3K/mTOR dataset. Reference molecules and their associated synthetic accessibility scores are displayed in Table 1. At the end of each generation, 100 molecules were randomly chosen among molecules with Tanimoto Similarity to the reference molecule higher than 0.8. Over those 100 molecules, 5 were randomly selected with $RScore3min \ge 0.5$, and 5 others with $RScore3min=0$, when it was possible. This led to a dataset of 30 molecules. Seven chemists were asked to blindly label molecules as either feasible (label 1) or not feasible (label 0). The final label of a molecule is the label given by the majority of chemists. This label is considered as a ground truth of synthetic accessibility. To assess the ability of the various synthetic scores to discriminate feasible and non feasible molecules, ROC-AUC is used as it is insensitive to the scale of the scores.

**Table 1:** Three molecules considered successively as reference molecule for the in-silico validation of the RScore. The values of the different synthetic scores for those molecules are also indicated

Name	Molecule	RA score	SC score	SA score	RScore3min	RSPred
Imatinib		1	4.996	2.33	1	0.72
Acetylsalicylic acid		1	1.59	1.58	1	0.83
Pi 3K/mTOR		0.975	4.995	3.56	0.6	0.55

#### Generations without any synthetic accessibility constraint

Ten generations were performed using the standardized Guacamol Benchmark. Molecules were generated over 20 epochs on each of the 10 MPO tasks of the Guacamol Benchmark, which are: Osimertinib MPO, Fexofenadine MPO, Ranolazine MPO, Perindopril MPO, Amlodipine MPO, Sitagliptin MPO, Zaleplon MPO, valsartan SMARTS, Deco Hop and Scaffold Hop. Each task is associated with an objective function, the description of each task can be found in the original paper [9].

**Fig. 1:**
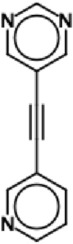
Imposed structure for Pi3K/mTOR generation

The next generation aimed at solving a lead optimization problem on the Pi3K/mTOR dataset. The constraints for this task were the Tanimoto similarity of ECFP4 fingerprints to the initial dataset, the Quantitative Estimate of Drug-likeness (QED) [35], and predicted Pi3K and mTOR pKi values. For Pi3K and mTOR pKi predicted values, two QSAR models were used as scorers during the ensuing generative procedure. Those were built using ECFP molecular representation with 4096 bits and with radius 4 for mTOR and 6 for Pi3K, molecular descriptors, and a ridge regression model. K-fold (K = 4) cross validation along with tree-structured Parzen Estimator was used to select the model and the fingerprints parameters. On a 20% hold out set, the R2 score of the Pi3K model and the mTor model are respectively 0.64 and 0.71. In addition to these scores, a filter was added to enforce a specific substructure within the generated molecules, corresponding to the following SMARTS pattern drawn in Fig. 1. The thresholds for each of the targets can be found in Table 2. The objective function associated with this task was the geometric mean of the five scoring functions as in Eq. 4.

3 $$\begin{aligned} smarts_1 = c1cncc(c1)C\#Cc1cncnc1 \end{aligned}$$

**Table 2:** Blueprint of the task Pi3K/mTOR

Criteria	Specification
Pi3K	> 7
mTOR	> 8.5
QED	> 0.5
Tanimoto similarity	> 0.5
contains structure $smarts_1$ ( Eq. 3)

4 $$\begin{aligned} Score\,(mol) = GeoMean\, \Big (score_1(mol),...,score_5(mol)\Big ) \end{aligned}$$

where:

5 $$\begin{aligned} GeoMean\,(x_1,..., x_n) = \left( \prod _{i=1}^{n}x_{i}\right) ^{\frac{1}{n}} = {\root n \of {x_{1}x_{2}\cdots x_{n}}} \end{aligned}$$

The prior model was the one trained on ChEMBL, then two steps of transfer learning were run on the Pi3K/mTOR dataset in order to remain within the applicability domain of the QSAR regressors. For the training part, the batch size was 1024, the learning rate 1e-3 and the generation run over 250 epochs.

#### Generations under synthetic accessibility constraint

Generations under synthetic constraint used the same parameters as described above, while incorporating a synthetic accessibility score in the reward. Compared to the previous generations, only the scoring function was changed, with the different synthetic scores added in the objective function as follows:

6 $$\begin{aligned} Score(mol) = GeoMean\, \Big (score_1(mol),...,score_k(mol), ScoreSynth(mol) \Big ) \end{aligned}$$

Where *ScoreSynth* can be any function that estimates synthetic accessibility: RA, SC, SA, RScore1min, or RSPred, on which a modifier function is applied. The function *GeoMean* is described in Eq. 5.

For each of the 10 Guacamol tasks, three generations were run with the ScoreSynth being successively SA score, RScore1min and RSPred. For the Pi3K/mTOR task, five generations were run with the ScoreSynth being successively RA score, SC score, SA score, RScore1min and RSPred. We conducted a post-processing analysis of the results using RScore3min. This score was considered the *ground truth* of synthetic accessibility, and the other synthetic scores were evaluated for their relevance as estimates of synthesizability as provided by RScore3min. In total, 35 generations under synthetic constraint were performed: 30 for the Guacamol Benchmark tasks, and five for the Pi3K/mTOR task.

#### Score modifiers

On each scoring function a modifier function is applied in order to normalize the score into the range [0, 1]. The modifier and its parameters are chosen based on the expected threshold for each target, and are well described in the literature [9]. The two modifiers used are MaxGaussian and MinGaussian:

- MinGaussian($\mu$, $\sigma$): the right half of a Gaussian function. Values smaller than $\mu$ are given full score, and values larger than $\mu$ decrease continuously to zero.
- MaxGaussian($\mu$, $\sigma$): the left half of a Gaussian function. Values larger than $\mu$ are given full score, and values smaller than $\mu$ decrease continuously to zero.
  The modifiers of the Guacamol tasks are specified in the original paper. The modifiers used in the Pi3K/mTOR task are described in Table 3.

**Table 3:** Modifiers used for the different scoring functions in the Pi3K/mTOR task

	Modifier
Pi3K	MaxGaussian (7, 1)
mTOR	MaxGaussian (8, 1)
QED	MaxGaussian (0.6, 0.13)
Similarity	MaxGaussian (0.75, 0.25)
RA Score	MaxGaussian (0.7, 0.2)
SC Score	MinGaussian (2.5, 0.4)
SA Score	MinGaussian (2.5, 0.4)
RScore1min	MaxGaussian (0.7, 0.2)
RSPred Score	MaxGaussian (0.7, 0.2)

## Results and discussions

In this section, first we compare the values of the different synthetic scores on molecules from the ChEMBL dataset, then we evaluate the performance of the RScore1min predictor (RSPred), then we analyze the results of the in-silico validation of the RScore3min, and finally we analyze the results of the different generations with and without synthetic constraints.

### Comparison of synthetic scores

**Fig. 2:**
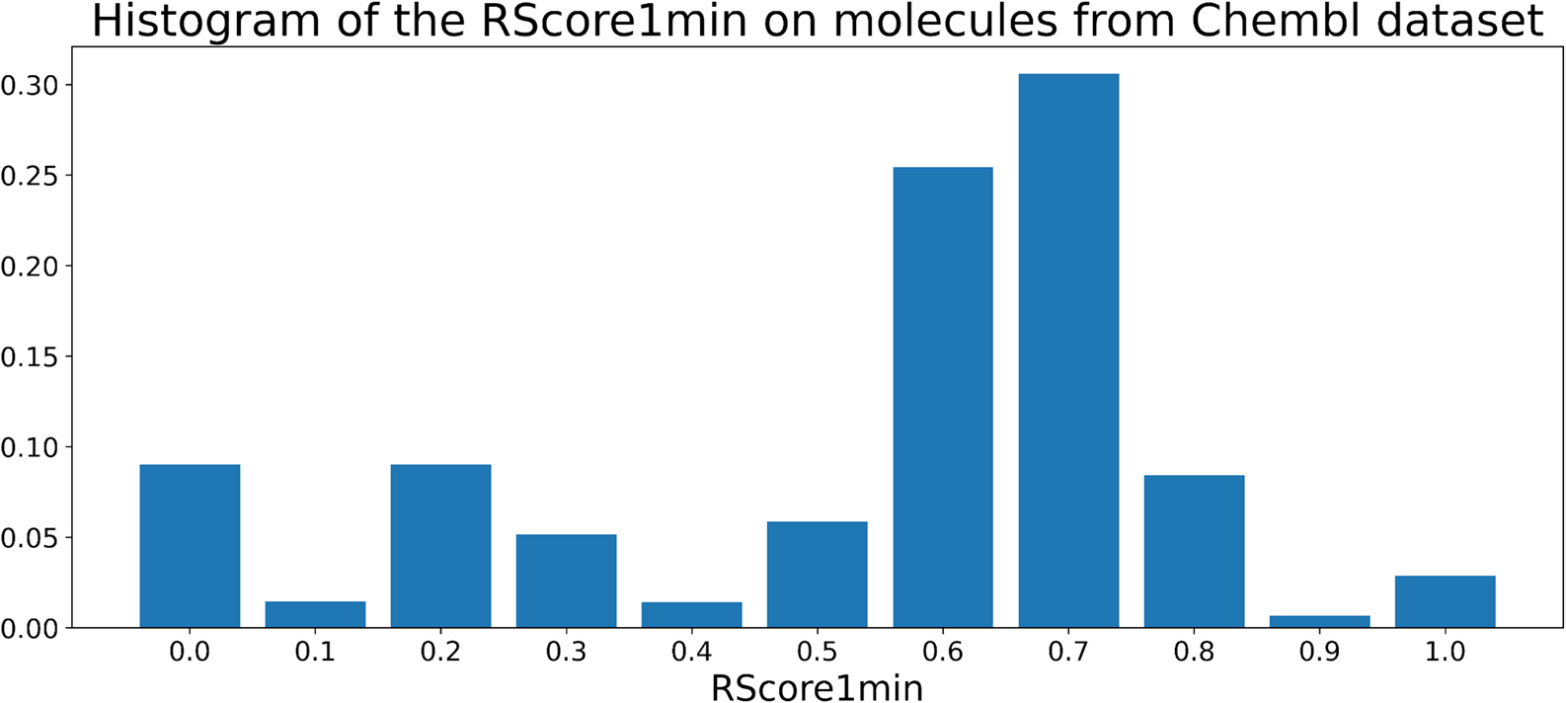
Normalized histogram of the RScore1min on molecules from Chembl dataset

**Fig. 3:**
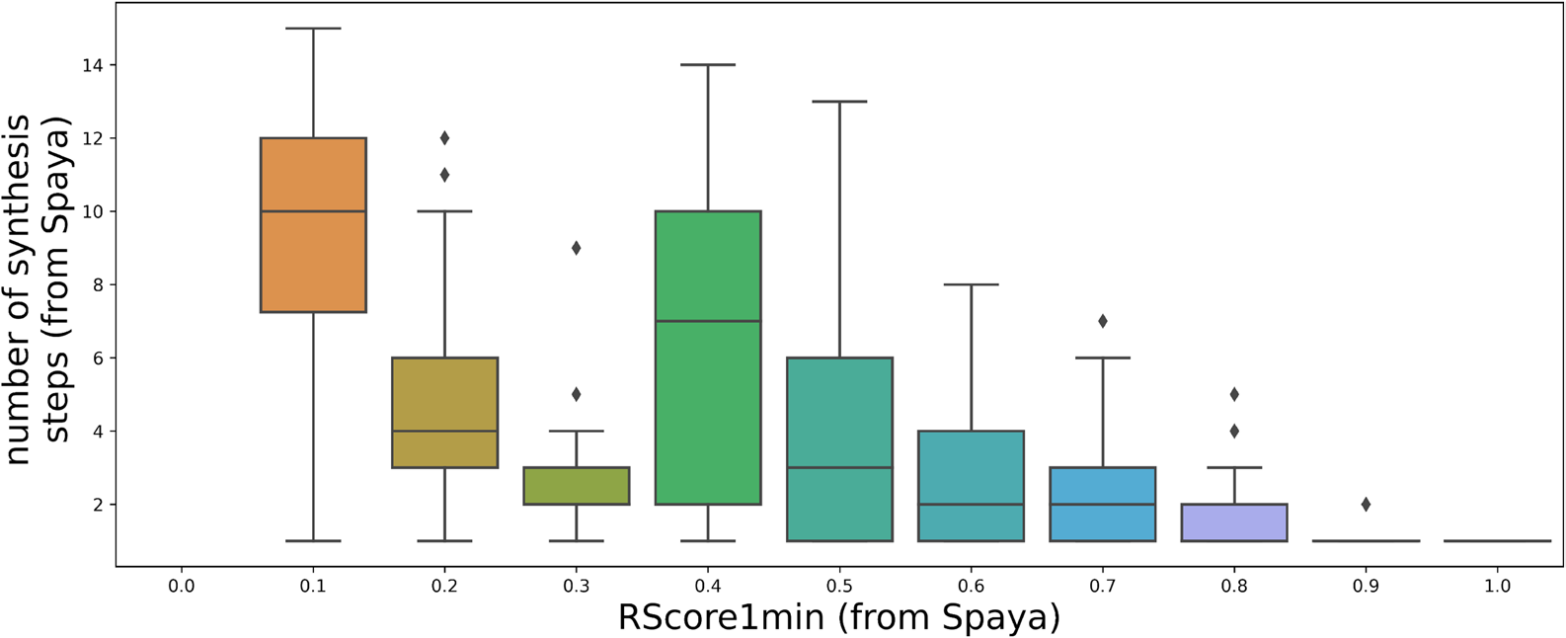
Correlation between the RScore1min and the number of synthetic steps given by Spaya API on a sample from Chembl dataset

Based on our experience and discussions with chemists, we consider the threshold for a good RScore to be 0.5, as molecules above this threshold often are considered “good enough” for chemists. The total distribution of the RScore1min on a sample of molecules from Chembl 24 is plotted on Fig. 2. It can be seen that around 66% of the sample have a good RScore1min ($\ge$ 0.5), that a significant part of the dataset is not solved by Spaya API and that a major mode around 0.7 is observed. The RScore is not directly interpretable but it takes into account the number of synthesis steps, which is a meaningful metric for chemists. The graph Fig. 3 is a plot of the correlation between the RScore1min and the number of steps of synthesis found by Spaya for the ChEMBL dataset sample.

It can be seen that few synthesis steps (fewer than 6) is a necessary condition for having a good RScore, though the contrary is not true. For instance a 2 steps route may have a bad score due to disconnections with low predicted probabilities. Indeed, the scoring function in Eq. 1 considers other elements than the number of steps to evaluate the route.

As previously discussed, existing literature scores designed to estimate synthesizability do not perform a full retrosynthetic analysis of the target molecule. Those scores were compared on a bench of molecules in order to analyze the extent to which they agreed with one another.

**Fig. 4:**
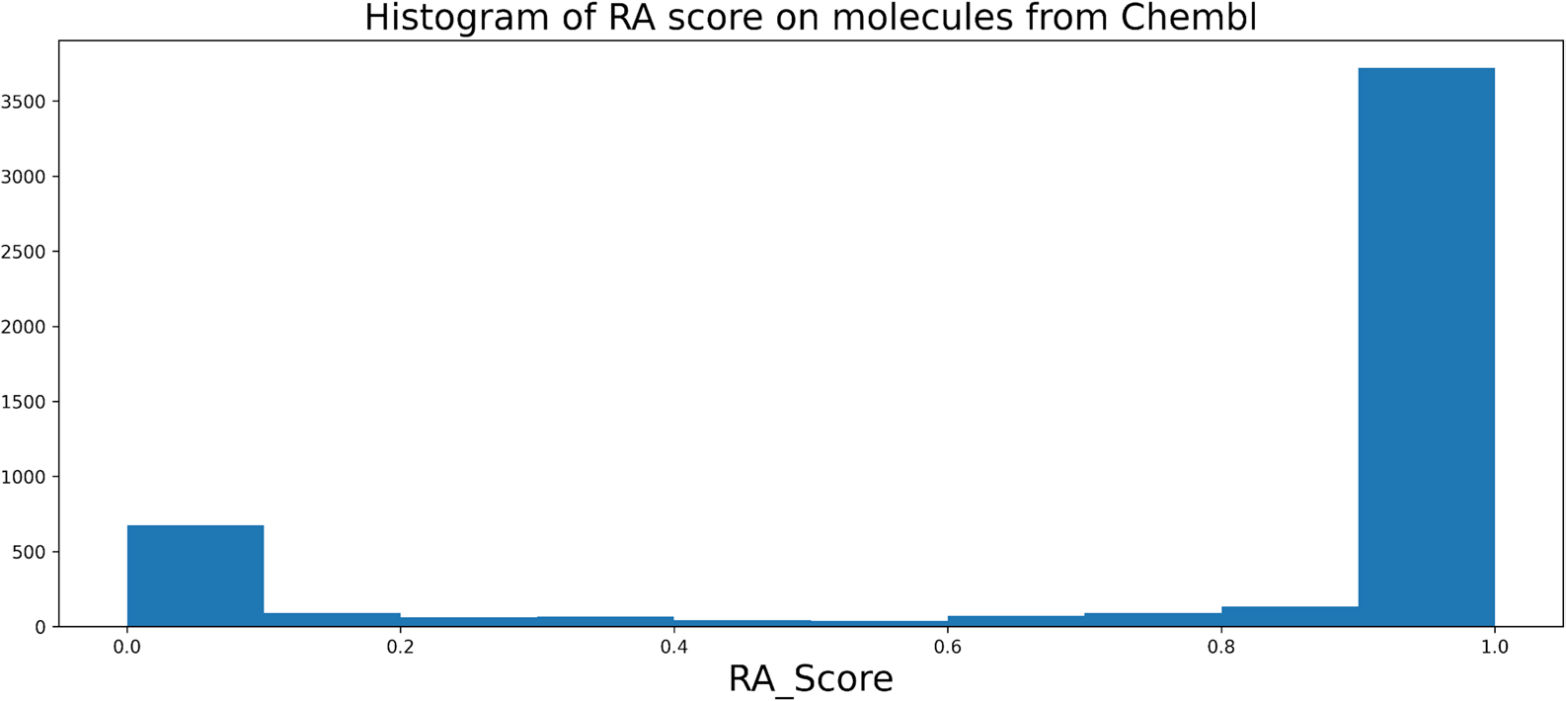
Histogram of RA score on the Chembl dataset

**Fig. 5:**
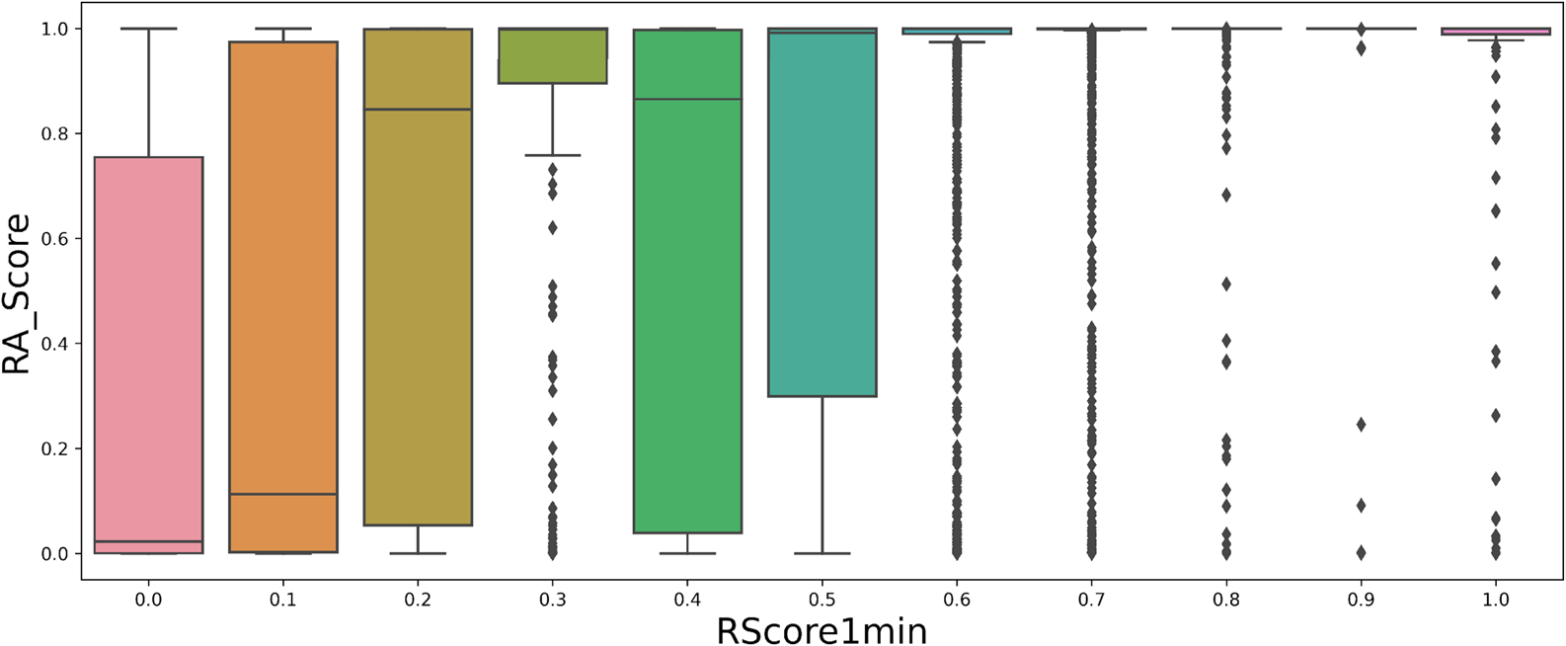
Correlation between RA score and RScore1min on Chembl dataset

**Fig. 6:**
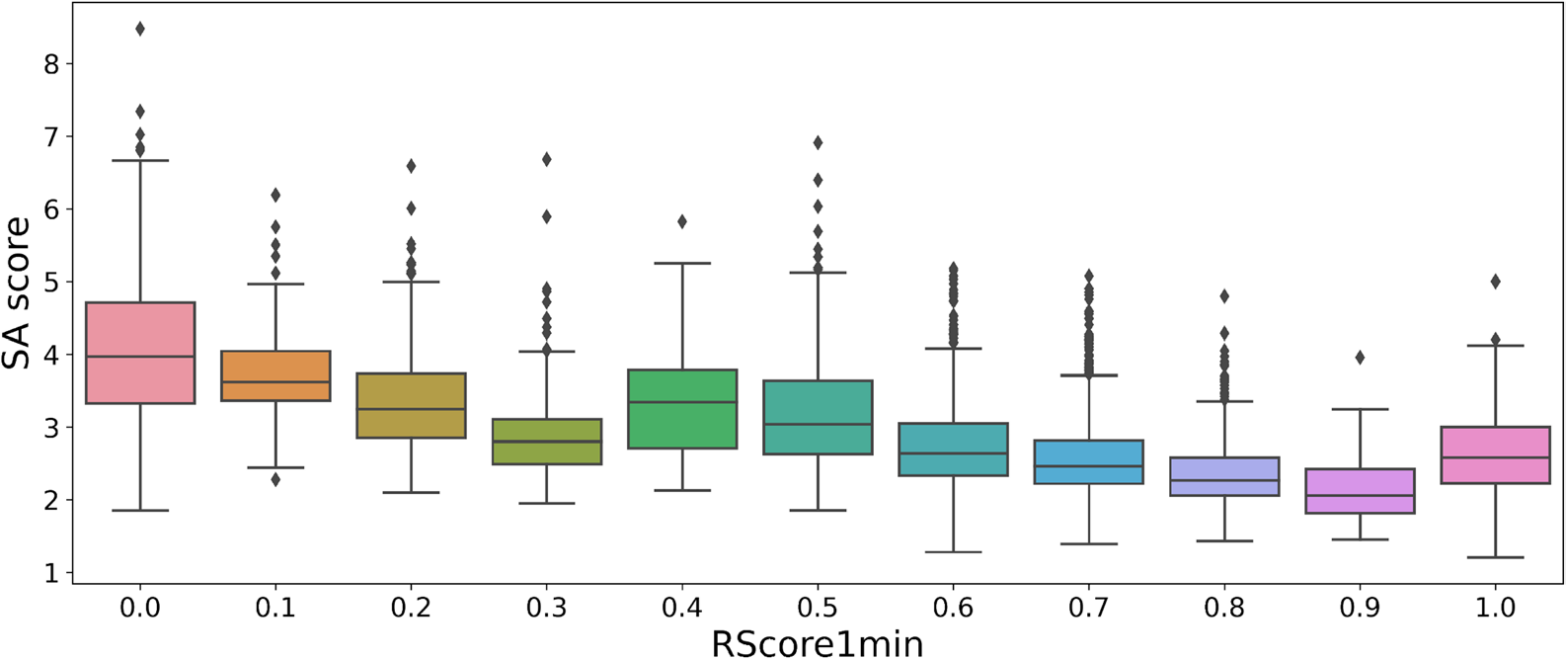
Correlation between SA score and RScore1min on Chembl dataset

**Fig. 7:**
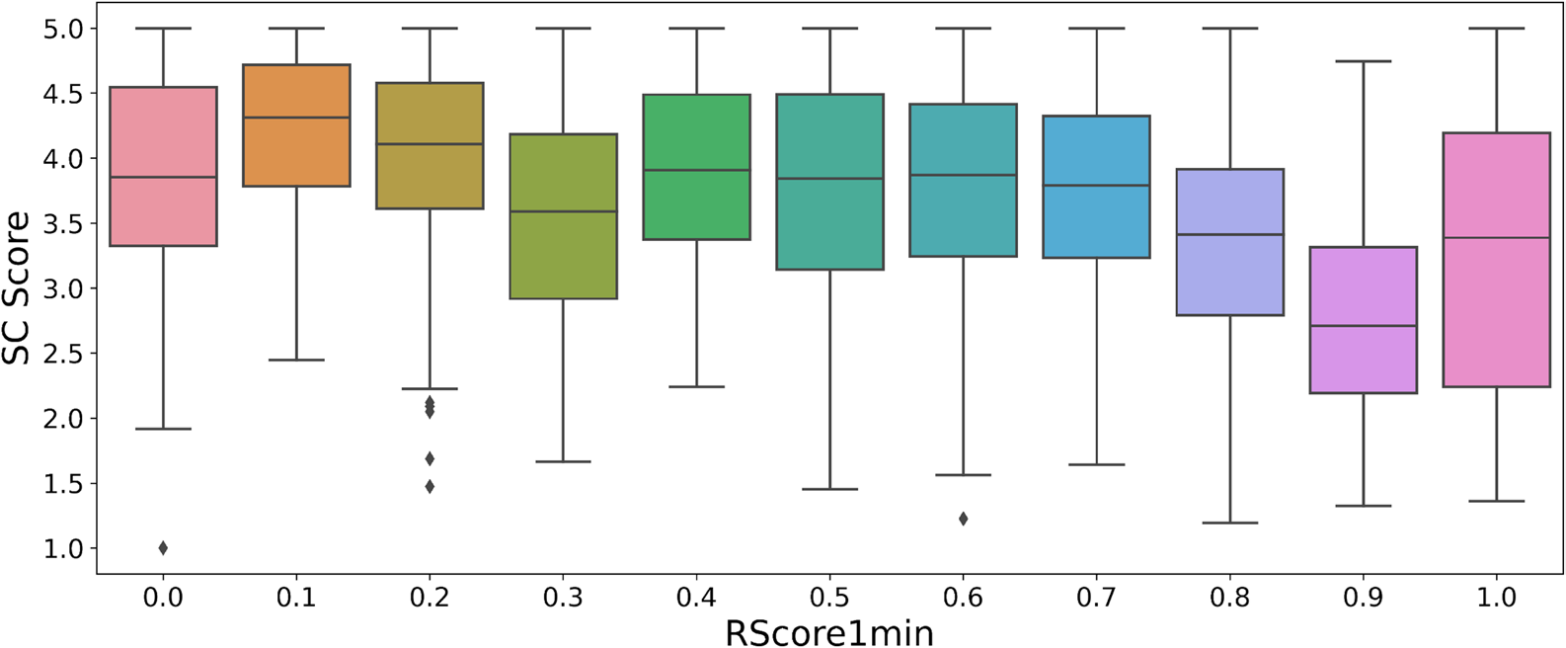
Correlation between SC score and RScore1min on Chembl dataset

On the ChEMBL dataset sample, the RA score (Fig. 4) often predicts a score of almost 1. Hence, this score is not useful to measure the difficulty of synthesis of feasible compounds (Fig. 5). This can be explained by the fact that the model computing the RA score was trained on a subset of ChEMBL. The SA score is significantly correlated to the RScore1min (Fig. 6). Having a good SA score seems to be a sufficient condition to have a good RScore, while the contrary is not true: molecules with complex fragments will often have a bad SA score, even if they are synthesizable. As an example, the molecules in Additional file 2: Fig. S3 contain original and complex fragments, but are easy to synthesize through Spaya. Finally, the SC score has no correlation at all with the RScore1min. (Fig. 7).

### RSPred

**Fig. 8:**
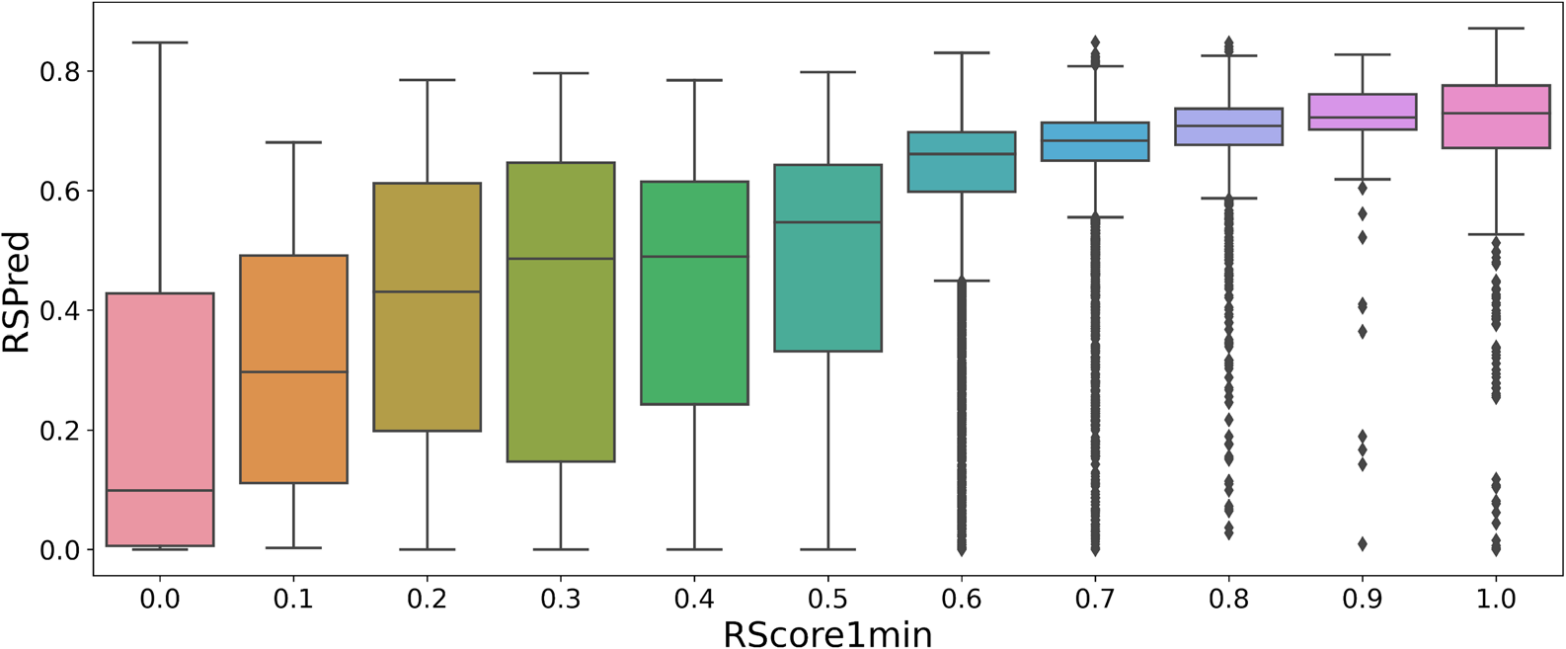
Correlation between the RScore1min and the values predicted from the neural network on a test set

In hopes of replacing a full retrosynthetic analysis with a prediction, a deep learning model was trained to predict the RScore1min obtained with Spaya-API. The performance of the neural network was evaluated on a hold out test set of the preprocessed Chembl dataset. The box plot of the predictions made by the neural networks with regards to the true RScore is shown in Fig. 8. With a Pearson correlation of 0.75, the results are quite satisfying. For this reason, the prediction of the neural network is considered as a new synthetic score, RSPred, which can be used as an additional synthetic constraint in molecular generations.

### Computing time

**Table 4:** Computing time per molecule for the different synthetic scores

Synthetic score	Time per molecule (ms)
RA score	28
SC score	241
SA score	2
RScore	40000
RSPred	1

Computing time is an essential attribute of a score as it may limit its usage on large scale data sets. Table 4 displays computing time estimates of the different synthetic scores. The RScore1min, being obtained through a full retrosynthesis, is by far the most time consuming score. Thanks to its scalability, Spaya-API accelerates RScore computation on batches of molecules. The prediction of the latter, RSPred, is the fastest score to compute, only 1ms per molecule, 40 000 time faster than the RScore1min. The SA score closely follows with 2ms per molecule, the RA score is one order of magnitude slower while the SC score is two orders of magnitude slower.

### In-silico validation of the RScore

The seven chemists asked to label the 30 selected molecules as feasible or not feasible all agreed with each other except two chemists on two molecules. Based on this, we can consider the synthetic accessibility label as being reliable. Over the 30 molecules, 12 were labelled as feasible by chemists. Details about the chemists labels and the synthetic scores values of the 30 molecules are given in the Supporting Information.

In Table 5 are displayed the ROC AUC [36] scores for binary prediction of synthetic accessibility, using the different synthetic scores. In the first three rows, ROC AUC is computed only on molecules selected from one generation, whereas in the last row all 30 selected molecules are included. The only synthetic score that is able to perfectly classify feasible/non feasible molecules is RScore3min.

We acknowledge that this experiment is only a partial validation of RScore, and so we invite any research group that would be interested in this work to contact us for further evaluation of our score.

**Table 5:** AUC to predict binary target feasible/non feasible over the 30 selected molecules, coming from 3 similarity constrained generations

Molecule name	RA score	SC score	SA score	RScore3min	RSPred
Imatinib	0.8	0.28	1	1	0.68
Acetylsalicylic acid	0.44	0	0.33	1	0.222
Pi3K/mTOR	0.76	0.4	0.96	1	0.48
Overall	0.41	0.201	0.63	1	0.335

### Evaluations of generations on 10 Guacamol tasks

**Table 6:** Average RScore3min and average reward of the top 100 molecules of the Guacamol generations without any synthetic accessibility constraint

Task name	Average RScore3min	% with RScore3min $\ge$ 0.5	Average reward
Amlodipine MPO	0.55	77	0.86
Deco HOP	0.66	97	0.99
Fexofenadine MPO	0.66	95	0.89
Osimertinib MPO	0.50	74	0.50
Perindopril MPO	0.59	94	0.59
Ranolazine MPO	0.39	50	0.39
Scaffold Hop	0.58	81	0.58
Sitagliptin MPO	0.60	82	0.60
Valsartan SMARTS	0.62	90	0.62
Zaleplon MPO	0.69	99	0.68

In this section, we evaluate the synthesizability of the most optimal generated molecules from the 10 Guacamol tasks. We then consider the impact of adding a synthetic constraint during the generation. The results are analyzed based on the initial objective functions as well as the synthetic accessibility as assessed by the RScore3min which is considered as the ground truth in this experiment.

Table 6 contains the reward and RScore3min (three minutes timeout) of the top 100 molecules generated without any synthetic constraint for each task. The ranking is performed based on the reward of each task. It should be noted that the top 100 molecules are already good in terms of synthetic accessibility and reward: an average of 98% of the optimized molecules are synthesizable (RScore3min above 0) according to Spaya-API, and a large majority even have a good RScore3min (above 0.5).

**Fig. 9:**
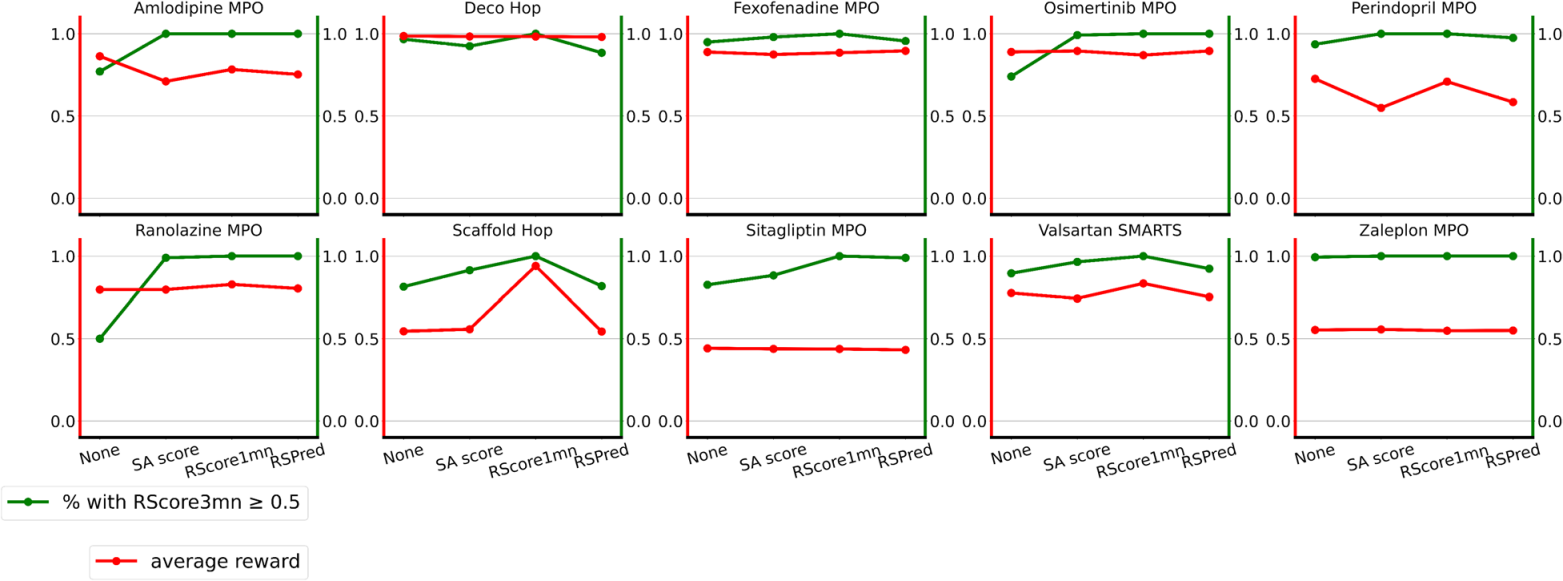
Reward and accessibility of the top 100 molecules for each task and with different synthetic constraints. The red line is the average reward (without the synthetic score) on the top 100 molecules of the generation. The green line is the percentage of the top 100 molecules with a RScore3min above or equal to 0.5

For each of the 10 benchmarks, in addition to the generations without any synthetic constraint, three generations were run with:

1. SA score constraint
2. RScore1min constraint
3. RSPred constraint

All those generations are compared based on two metrics: the average RScore3min on the top 100 molecules, and the average reward on the top 100 molecules, where the top 100 are selected based on their score on the initial objective function. The plots in Fig. 9 summarize the results of the different generations. As previously stated, even without any synthetic constraint in the scoring function, the top 100 molecules of these generations have a reasonably good RScore3min. The SA score constraint improves the RScore3min of the top molecules, and the RScore1min and RSPred constraints improve it even more. Importantly, the reward is generally not degraded by the synthetic score constraint.

These tasks may be insufficient to evaluate the impact of adding a synthetic constraint during generation, due to their relative ease. Indeed, we have observed that in real-life drug design projects the synthetic accessibility of the generated molecules is usually a more prominent issue when the optimization tasks are harder to solve. We reason that this occurs because when the generator struggles to find a solution it designs more and more awkward structures to satisfy the goal criteria, resulting in molecules which are likely not synthesizable. Hence, the generation under synthetic constraint is a potential solution as it keeps orienting the generative model in a chemical space of feasible molecules. This is the motivation behind the “Pi3K/mTOR experiment”: it is a more realistic model of a real-life drug design project and reflects better the impact of using a synthetic accessibility constraint during molecular generation.

### Evaluations of generations performed on the Pi3K/mTOR dataset

This task is a generation around a library of 463 structurally homogeneous Pi3K and mTOR inhibitors. The objective and targets can be found in Table 2. This dataset serves as a simplified proxy for a real life MPO in a lead optimization project with four objectives to be optimized (Table 2). Six generations were run based on this dataset: one without any synthetic score constraint, and five with synthetic score constraints (RA, SC, SA, RScore1min, and RSPred). When looking at the evolution of each component of the score among epochs in Additional file 2: Fig. S4, it can be noted that the reward increases and saturates systematically around epoch 60.

#### Synthetic accessibility of generated molecules in the blueprint

Here, the main metric to evaluate the quality of a generation method is the number of generated molecules validating all the constraints which also have a good RScore3min. The number of generated molecules for each generation (Table 7) is roughly constant (± 3%), but the number of unique molecules is more variable.

**Table 7:** Number of molecules generated for each generation; the first column indicates which synthetic score constraint was used. The last column corresponds to the number of generated molecules in the blueprint

Synthetic constraint	n Molecules	n Unique molecules
None	80399	16085
RAscore	80663	14509
SCscore	80291	14635
SAscore	78612	12775
RScore1min	83215	11081
RSPred	79861	12703

**Fig. 10:**
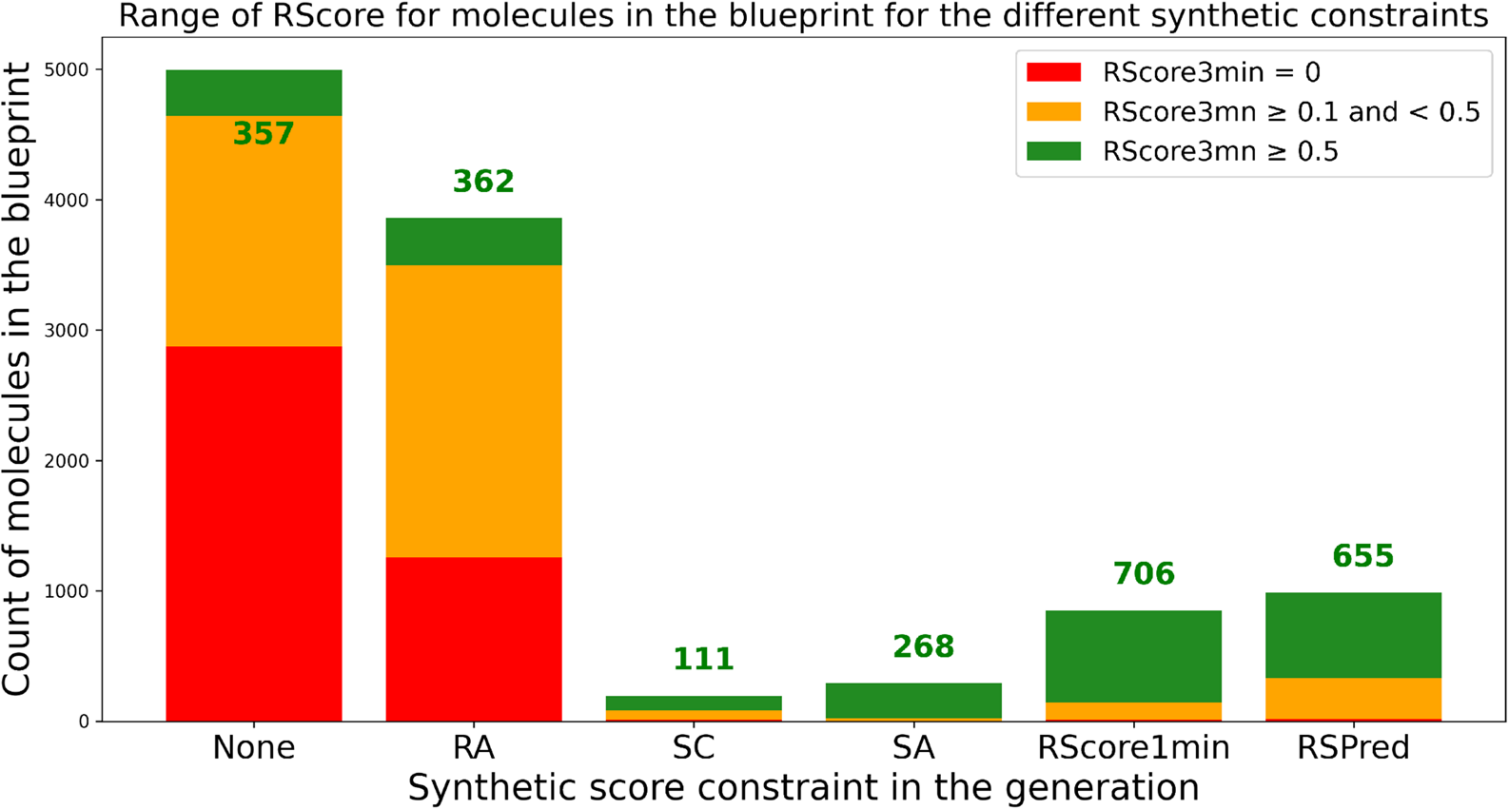
Number of molecules in the blueprint for each generation, with indication on their RScore3min range

A molecule is said to be in the blueprint when the computed value of each objective is in the desired range. The graph Fig. 10 shows for each of the five generations the number of molecules in the blueprint and their RScore3min range.

First, we observe that the generation without synthetic constraint and the one with the RA constraint both contain a high percentage of non-synthesizable molecules (as assessed by the RScore3min), which would be problematic in a real-life project. In the other generations, almost all molecules in the blueprint are synthesizable, however the generation with SC constraint produced very few molecules in the blueprint, and the generation with SA constraint significantly less molecules (268) compared to the generation with RScore1min constraint (706) and the generation with RSPred constraint (655). The generation with the RScore1min constraint gave, unsurprisingly, the best results, as the RScore1min is highly correlated with the RScore3min which is used as the ground truth of synthesizability in this experiment. The RSPred generation produced almost as many easy to make molecules as the RScore1min generation. It seems that the RSPred constraint was sufficient to lead the generative algorithm towards the generation of synthesizable molecules (Fig. 10).

**Table 8:** Comparison of the chemist score over the top 10 molecules for each generation.

Synthetic contsraint	Chemist score	Std chemists score
None	0	0
SCscore	0.58	0.20
RAscore	0.61	0.20
SAscore	0.63	0.15
RScore1mn	0.63	0.16
RSPred	0.63	0.16

The chemist score for a molecule is the average of labels 0/1 given by 7 chemists to assess the synthetic accessibility. The first column indicates which synthetic accessibility constraint was used in this generation. The last column show the standard deviation of the scores given by the chemists

To achieve a less biased evaluation of the generative AI model, we employed an alternative method to assess the synthetic accessibility of the generated molecules. As detailed in section In-silico validation of the RScore, while it is difficult to definitively establish a ground truth for this metric, we found that chemists’ intuition provided a valuable means of evaluating synthetic accessibility. In this regard, we asked the same panel of seven chemists to evaluate the synthetic accessibility of the top 10 molecules (based on the reward) generated in each experiment. The chemist score for a molecule was determined by calculating the average of labels 0/1 assigned by the panel. As presented in Table 8, the application of any synthetic accessibility constraint led to a significant improvement in the average chemist score, with RScore1mn, RSPred, and SAscore yielding the best results. The standard deviation of the 7 scores given by chemists remained below 0.2 for all experiments. Overall these findings underscore the potential of incorporating synthetic accessibility constraints in generative AI models for drug design. The top 10 molecules per generation scored by chemists are shown in Additional file 2: Figs. S5–S10.

#### Diversity of generated molecules

**Table 9:** Some statistics about the molecules in the blueprint for the 6 generations

Synth constraint	Count	Average RScore3min	Feasible	Good RScore3min	Standard Murckos	Generic Murckos	Unique
None	5005	0.08	1959	282 (6%)	59 (21%)	36 (13%)	34
RA	3574	0.11	2660	360 (10%)	79 (22%)	47 (13%)	64
SC	211	0.35	202	127 (60%)	19 (15%)	14 (11%)	64
SA	311	0.56	311	286 (92%)	40 (14%)	31 (11%)	145
RScore1min	850	0.49	843	706 (83%)	69 (10%)	46 (7%)	314
RSPred	985	0.46	971	655 (66%)	104 (16%)	73 (11%)	357

Standard Murckos and Generic Murckos: the number of different Murcko. Feasible/good RScore: number of molecules with RScore $>0$ / $\ge 0.5$. Unique: # of molecules that are only in this generation (and not in any of the other five). All the columns after ’good RScore3min’ refer to the molecules in the blueprint with a good RScore3min

Table 9 displays information about the generated molecules which met all the objectives described in Table 2. It shows that the generations under RScore1min or RSPred constraint enabled to find two to four times more easy-to-make molecules than the other generation methods. We notice that the generations with no synthetic constraint and under RA constraint produced more molecules in the blueprint but few of those had a good RScore3min, while the generations under SC and SA constraints produced less molecules in the blueprint, and less molecules in the blueprint with a good RScore3min.

To evaluate the diversity of the generated molecules, we computed the number and percentage (in parenthesis) of Murcko scaffolds and generic Murckos scaffolds [37] among the molecules in the blueprint with a good RScore3min. We observe that diversity is not significantly different among the different methods, though RScore1min and RSPred generations did produce more scaffolds than the other methods. The RScore1min and RSPred methods also generated a significant number of compounds which could not be found with the other methods (more than 300). This seems to imply that the synthetic constraint in the RScore and RSPred generations led the generative algorithm to explore a different area of the chemical space, identifying solutions meeting both the blueprint and the synthetic accessibility constraint that could not be found with other methods.

**Fig. 11:**
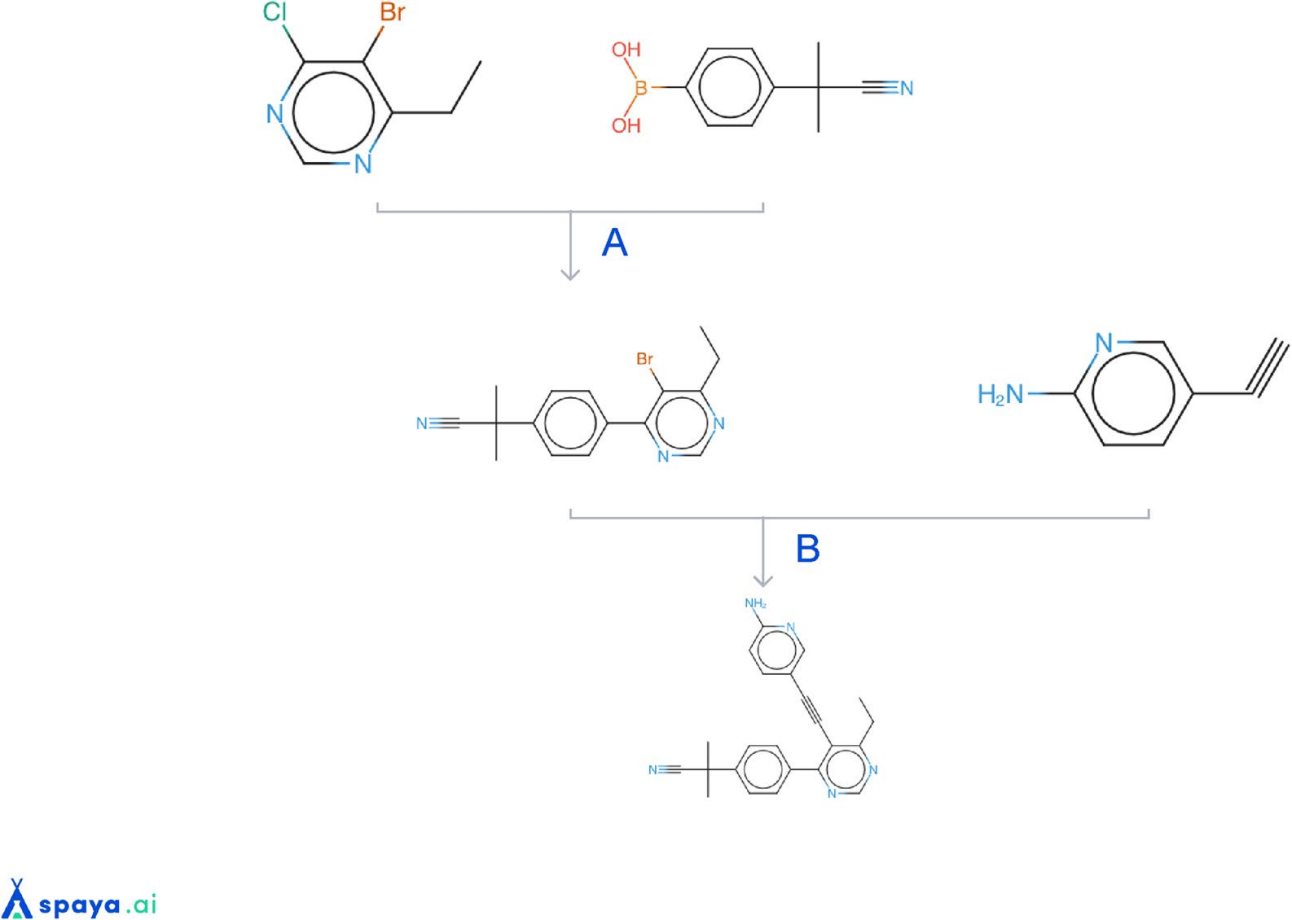
Example of a synthesis route obtained by Spaya

In order to illustrate the output of those generations, we show in Additional file 2: Figs. S3–S10 the top 10 molecules of each generation, where the selection process was the following: after filtering on the molecules validating the four thresholds, the top 10 molecules regarding the optimized synthetic score were selected. In Additional file 2: Fig. S11 are shown some molecules generated under RScore1min constraint that may be interesting according to a chemist. An example of a synthetic route can be found in Fig. 11. This route contains 3 commercial compounds and two synthesis steps.

## Discussion

In this paper, we introduced the RScore, a new in silico score of the synthetic accessibility of molecules, which is meant to be used to assess the synthetic accessibility of molecules designed by generative algorithms. Unlike other synthetic accessibility scores, the RScore is built based on the results of a full retrosynthetic analysis. As it is slower and more expensive to compute, it is important to understand how it compares to alternative scores. The experiment we performed regarding the *in silico* validation of the RScore showed that it outperformed the other synthetic accessibility scores, but more importantly, it behaved as a global score, in the sense that its value remains consistent over a broad chemical space. Indeed, as the RScore links an input molecule to a large set of building blocks, it successfully ranks molecules coming from heterogeneous chemical series, whereas the other benchmarked scores do not generalize in the same way. Conversely, the SA Score, which was the second best score in this experiment, performed well for each separate generation, i.e., it was able to assess the relative complexity of molecules similar to each other, but it failed to discriminate the synthetic accessibility of molecules with very different chemical structures. As a result, the RScore may be well fit for assessing the synthetic accessibility of highly diverse sets of generated molecules, such as in hit discovery/scaffold hopping scenarios. Complexity and synthetic accessibility are two different notions, and although they are usually correlated, complex molecules can be feasible and simple molecules can be unfeasible. It is easy to find complex molecules that are synthetically easy to make: a simple reaction using two complex building blocks may lead to an easily accessible and highly complex molecule. Because of the way it is built, the RScore is therefore better suited to assess synthetic accessibility, whereas the SA score is better suited to assess molecular complexity. Another important feature of the RScore is that it can be customized to a specific context both in terms of available reactions and available building blocks. Indeed, changing the reaction space or the catalogue of building blocks may give different RScore results for a given set of molecules (Data not shown). This feature can be very useful in real-life projects, however it makes the formal assessment of the RScore vs other scores more complex.

As the RScore is intended to be used in the context of generative chemistry, not only to triage the molecules produced by generative algorithms but also to guide generative algorithms, it is important to understand how the introduction of a synthetic constraint influences the output of molecular generations in various tasks. We showed that for simple Guacamol tasks, the impact of introducing a synthetic constraint is limited as most of the molecules generated have good synthetic accessibility scores. However, this may not be the case for more complex generative tasks incorporating a larger number of objectives difficult to combine, which are more representative of real-life projects. This was the objective of the Pi3K/mTOR experiment, which is closer to a real-life MPO scenario. In that experiment, introducing a synthetic accessibility constraint during the generation proved to have a major impact on the synthesizability of the generated molecules, and RScore1min appeared to be the best synthetic accessibility score in that context, as it outperformed the other methods by enabling the generation of a high number of molecules in the blueprint with good synthetic accessibility. We also showed that the RSPred, a neural network trained to predict the RScore, is a good proxy of the RScore1min with a much lower computational cost, making it very interesting to use as a substitute of the slower and more costly RScore1min in generative chemistry pipelines. Among the remaining scores which were evaluated as synthetic constraints during molecular generation, the SA score was the only one which produced mostly synthesizable results, though the generation under SA constraint still produced less than half as many molecules as the generation under RScore1min or RSPred constraint. The other synthetic constraints did not perform well in the experiment: the RA score has a poor precision, meaning that among the molecules well scored by RA score, very few actually have a good RScore3min. When used as a constraint in the reward of a generation, most molecules get a high RA score, so the generator cannot be optimized towards easier to make molecules. The SC score has no correlation to the RScore3min, so it comes as no surprise that generation under SC score constraint fails to optimize the RScore3min during the generation and gives poor results. The prior used in this study was trained on ChEMBL24 [20], which included approximately 30% of molecules with a bad RScore (Fig. 2). We did not assess if using a dataset with only synthesizable molecules to train the prior would result in more synthesizable generated molecules. At the same time, reducing the size and chemical diversity of the initial set might also have an impact on the ability to find solutions to the MPO problem. This will be the topic of future works.

This work has several limitations: first, we acknowledge that the validation of the RScore as a good synthetic accessibility score has been performed on a small number of molecules (30), and the validation dataset can be expanded. For such purpose, it would be a useful contribution to the community to develop and make available benchmark datasets of generated molecules with synthetic accessibility labels assigned by chemists which could be used to assess the value of synthetic feasiblity scores in a generative chemistry context. Second, the example use case with RScore1min as a synthetic constraint during complex MPO generation was conducted on only one dataset. The reason for this is the difficulty to find adequate publicly available datasets which are representative of the challenges of multi-parametric optimization in real-life lead optimization projects. We found the MPO datasets and tasks available in the Guacamol benchmark trivial to solve, and therefore not adequate for our purpose. Additional work has been performed by Iktos on other MPO datasets (not disclosed), showing similar results and conclusions aligned with the Pi3K/mTOR experiment. Third, in all our experiments of generations under synthetic constraint, we consider the “ground truth” of synthetic accessibility to be the RScore3min, which creates a strong bias, since by construction the RScore1min is strongly correlated to the RScore3min. It is therefore not surprising that generations under RScore1min perform better generating molecules with good RScore3min scores as compared to other synthetic constraints. The reason for such choice in the design of our experiments is that there is no known computational score which could be considered as an objective measure of the synthetic accessibility of molecules. Additionally, the number of molecules resulting from a molecular generation experiment made it impractical to ask chemists to assess them by hand. The absence of an absolute ground truth of what is synthetic accessibility, the fact that chemists themselves may sometimes disagree on the ease of synthesis of a given molecule, and the fact that the synthetic accessibility of a molecule may be highly dependent on the building blocks and reactions available, which themselves vary over time, all contribute to making a completely rigorous and objective analysis close to impossible. Despite these intrinsic limitations, we reiterate what we believe is the major advantage of the RScore, i.e., the fact that it derives from the output of a real retrosynthetic analysis. In our experience using the RScore on a daily basis to assess the synthetic accessibility of molecules produced by generative algorithms, alongside traditional medicinal chemistry analysis, we usually observe a good agreement between the RScore and the chemists’ opinions.

## Conclusion

Molecular generation methods are known to produce unrealistic structures which can be impossible to synthesize, and known synthetic scores often fail to address that issue. In this paper, we introduce a new synthetic accessibility score, RScore, derived from Spaya [19], a data-driven synthetic planning software developed by Iktos. The main advantage that distinguishes RScore from other synthetic scores is that it is computed from the output of a full retrosynthetic analysis performed by Spaya. We show on a limited validation dataset that the RScore correlates very well, and better than other synthetic accessibility scores, with the assessment made by seven chemists regarding the synthetic accessibility of generated molecules. An important feature of the RScore is that it can be customized to better reflect the synthetic constraints of real life situations: the user can impose intermediate products to be in the routes, limit the number of steps, and customize the list of starting materials or the reaction space.

We also show that introducing the RScore as a synthetic constraint in a complex MPO molecular generation task which tends to produce synthetically infeasible molecules enables the design of synthetically accessible molecules by the generative algorithm, whereas other known synthetic scores do not perform as well. Conversely, for relatively simple molecular generation tasks, applying the the RScore as a post-processing filter seems to be sufficient, as most generated molecules have good synthetic accessibility. This warrants the use of a synthetic constraint, ideally RScore3min, in generative chemistry pipelines when trying to solve complex MPO challenges.

The computational complexity of the RScore is a limitation, hence a predictor of the RScore, RSPred, was built in order to accelerate the scoring. In a relatively difficult MPO task where generations under constraint of different synthetic scores were compared, RSPred constrained generations gave the best results second to RScore. We feel that RSPred strikes a good compromise, producing good results while being fast to compute. However, just as any machine learning model, the RSPred predictor has an applicability domain, in this case that of the ChEMBL dataset on which it was trained. Although the RSPred results were good in our experiment, this may be explained by the fact that the initial chemical space was already within the applicability domain of the predictor. In other cases, when the initial chemical space is far from ChEMBL, the predictor may have poor results and might lead the generation to an area of false positives. To address that issue, a preliminary fine tuning of the predictor on the chemical space of the generation might be helpful, if not necessary, to make sure the predictor’s performance is still sufficient. Investigations are ongoing regarding that topic.

### Supplementary Information

**Additional file 1.** Synthetic accessibility scores of the 30 generated molecules in the experiment of in-silico validation of rscore.

**Additional file 2: Figure S1.** Overview of the retrosynthesis technology behind Spaya. **Table S1.** List of building blocks providers used by Spaya.ai. **Figure S2.** Impact of the timeout on the RScore, for 1000 molecules sampled fromChEMBL24. **Figure S3.** Example of molecules with a bad SA score (> 3.5) but a good RScore (> 0.4). **Figure S4.** Evolution of 4 scoring functions : PI3K, mTOR, similarity, QED among epochsfor 6 different generations around PI3K/mTOR dataset. **Figure S5.** Top 10 molecules from PI3K/mTOR generation without any synthetic constraint. **Figure S6. ** Top 10 molecules from PI3K/mTOR RA score constrained generation. **Figure S7.** Top 10 molecules from PI3K/mTOR SC score constrained generation. **Figure S8.** Top 10 molecules from PI3K/mTOR SA score constrained generation. **Figure S9.** Top 10 molecules from PI3K/mTOR RScore constrained generation. **Figure S10.** Top 10 molecules from PI3K/mTOR RSPred constrained generation. **Figure S11.** Molecules generated during RScore constrained generation.

## Data Availability

A GitHub project contains all the scripts associated to the experiments [25], including generation under synthetic constraint. The dataset Pi3K/mTOR is in the git, and the ChEMBL 24 dataset can be downloaded following the link [21]. All the scoring functions are also implemented in the GitHub project, including pytorch model RSPred and its weights. Spaya (https://spaya.ai/) is a Software as a Service (SaaS) platform freely accessible on the web and running on Iktos’s secure Virtual Private Cloud (VPC) on Amazon Web Services (AWS). Iktos has packaged this high-throughput synthetic access scoring technology inside an API (Application Programming Interface) that allows customers to access the technology through various channels and tools (python code, scripts, jupyter notebook...). Spaya-API is available on demand under licence.

## References

[1] Segler MHS, Kogej T, Tyrchan C, Waller MP (2018). Generating focused molecule libraries for drug discovery with recurrent neural networks. ACS Cent Sci.

[2] Perron Q, Mirguet O, Tajmouati H, Skiredj A, Rojas A, Gohier A, Ducrot P, Bourguignon MP, Sansilvestri-Morel P, Do Huu N (2021). Deep generative models for ligand-based de novo design applied to multi-parametric optimization. ChemRxiv.

[3] Olivecrona M, Blaschke T, Engkvist O, Chen H (2017). Molecular de novo design through deep reinforcement learning. J Cheminf.

[4] Gómez-Bombarelli R, Wei JN, Duvenaud D, Hernández-Lobato JM, Sánchez-Lengeling B, Sheberla D, Aguilera-Iparraguirre J, Hirzel TD, Adams RP, Aspuru-Guzik A (2018). Automatic chemical design using a data-driven continuous representation of molecules. ACS Cent Sci.

[5] Sattarov B, Baskin II, Horvath D, Marcou G, Bjerrum EJ, De Varnek A (2019). Novo molecular design by combining deep autoencoder recurrent neural networks with generative topographic mapping. J Chem Inf Model.

[6] Gao K, Nguyen DD, Tu M, Wei G-W (2020). Generative network complex for the automated generation of drug-like molecules. J Chem Inf Model.

[7] Winter R, Montanari F, Steffen A, Briem H, Noé F, Clevert D-A (2019). Efficient multi-objective molecular optimization in a continuous latent space. Chem Sci.

[8] Renz P, Van Rompaey D, Wegner JK, Hochreiter S, Klambauer G (2019). On failure modes in molecule generation and optimization. Drug Discov Today Technol.

[9] Brown N, Fiscato M, Segler MH, Vaucher AC (2019). GuacaMol: benchmarking models for de novo molecular design. J Chem Inf Model.

[10] Bradshaw J, Paige B, Kusner MJ, Segler MHS, Hernández-Lobato JM (2019). A model to search for synthesizable molecules. CoRR.

[11] Bradshaw J, Paige B, Kusner MJ, Segler MHS, Hernández-Lobato JM (2020). Barking up the right tree: an approach to search over molecule synthesis DAGs. CoRR.

[12] Liu C, Korablyov M, Jastrzebski S, Wlodarczyk-Pruszynski P, Bengio Y, Segler MHS (2020). RetroGNN: approximating retrosynthesis by graph neural networks for de novo drug design. CoRR.

[13] Gao W, Coley CW (2020). The synthesizability of molecules proposed by generative models. J Chem Inf Model.

[14] Cumming J, Davis A, Muresan S, Haeberlein M, Chen H (2013). Chemical predictive modelling to improve compound quality. Nat Rev Drug discov.

[15] Coley CW, Rogers L, Green WH, Jensen KF (2018). SCScore: synthetic complexity learned from a reaction corpus. J Chem Inf Model.

[16] Ertl P, Schuffenhauer A (2009). Estimation of synthetic accessibility score of drug-like molecules based on molecular complexity and fragment contributions. J Cheminf.

[17] Thakkar A, Chadimová V, Bjerrum EJ, Engkvist O, Reymond J-L (2021). Retrosynthetic accessibility score (RAscore)—rapid machine learned synthesizability classification from AI driven retrosynthetic planning. Chem Sci.

[18] Genheden S, Thakkar A, Chadimová V, Reymond JL, Engkvist O, Bjerrum E (2020). AiZynthFinder: a fast, robust and flexible open-source software for retrosynthetic planning. J Cheminf.

[19] IKTOS Website Spaya (2023) https://spaya.ai/. Accessed 21 Feb 2023

[20] Mendez D (2018). ChEMBL: towards direct deposition of bioassay data. Nucleic Acids Res.

[21] Post-processed ChEMBL datasets. https://figshare.com/ projects/GuacaMol/56639. Accessed 20 Nov 2018

[22] Engelman JA (2009). Targeting PI3K signalling in cancer: opportunities, challenges and limitations. Nat Rev Cancer.

[23] Carnero A (2009). Novel inhibitors of the PI3K family. Expert Opin Investig Drugs.

[24] Liu P (2009). Targeting the phosphoinositide 3-kinase pathway in cancer. Nat Rev Drug Discov.

[25] Iktos GitHub containing the code reproducing the paper. (2023) https://github.com/iktos/generation-under-synthetic-constraint/. Accessed 28 Feb 2023

[26] RA score repository (2023) https://github.com/reymond-group/RAscore. Accessed 28 Feb 2023

[27] SC score repository (2023) https://github.com/connorcoley/scscore. Accessed 28 Feb 2023

[28] SA score repository (2023) https://github.com/EricTing/SAscore. Accessed 28 Feb 2023

[29] Ioffe S, Szegedy C (2015) Batch normalization: accelerating deep network training by reducing internal covariate shift. https://arxiv.org/abs/1502.03167

[30] Srivastava N, Hinton G, Krizhevsky A, Sutskever I, Salakhutdinov R (2014). Dropout: a simple way to prevent neural networks from overfitting. J Mach Learn Res.

[31] Kingma D, Ba J (2014) Adam: a method for stochastic optimization. In: International Conference on Learning Representations PMC13460018 42583277

[32] BenevolantAI Guacamol github. (2023) https://github.com/BenevolentAI/guacamol/. Accessed 3 Mar 2023

[33] Myung IJ (2003). Tutorial on maximum likelihood estimation. J Math Psychol.

[34] Lamb A, Goyal A, Zhang Y, Zhang S, Courville A, Bengio Y (2016) Professor forcing: a new algorithm for training recurrent networks. https://arxiv.org/abs/1610.09038 [stat.ML]

[35] Bickerton R, Paolini G, Besnard J, Muresan S, Hopkins A (2012). Quantifying the chemical beauty of drugs. Nat Chem.

[36] Melo F (2013). Encyclopedia of systems biology.

[37] Bemis GW, Murcko MA (1996). The properties of known drugs. 1. Molecular frameworks. J Med Chem.

