# Supplementary material for: Integrating synthetic accessibility with AI-based generative drug design
Source: J Cheminform. 2023 Sep 19;15:83. doi: 10.1186/s13321-023-00742-8 (PMC10507964; doi:10.1186/s13321-023-00742-8)
Supplement: Supplementary file 2 — Additional file 2: Figure S1. Overview of the retrosynthesis technology behind Spaya. Table S1. List of building blocks providers used by Spaya.ai. Figure S2. Impact of the timeout on the RScore, for 1000 molecules sampled fromChEMBL24. Figure S3. Example of molecules with a bad SA score (> 3.5) but a good RScore (> 0.4). Figure S4. Evolution of 4 scoring functions : PI3K, mTOR, similarity, QED among epochsfor 6 different generations around PI3K/mTOR dataset. Figure S5. Top 10 molecules from PI3K/mTOR generation without any synthetic constraint. Figure S6. Top 10 molecules from PI3K/mTOR RA score constrained generation. Figure S7. Top 10 molecules from PI3K/mTOR SC score constrained generation. Figure S8. Top 10 molecules from PI3K/mTOR SA score constrained generation. Figure S9. Top 10 molecules from PI3K/mTOR RScore constrained generation. Figure S10. Top 10 molecules from PI3K/mTOR RSPred constrained generation. Figure S11. Molecules generated during RScore constrained generation. [file 13321_2023_742_MOESM2_ESM.pdf]

Supporting Information
Integrating synthetic accessibility with AI-based generative drug design

Maud Parrot[1], Hamza Tajmouati[1], Vinicius Barros Ribeiro da Silva[1], Brian Ross Atwood[1], Robin Fourcade[1], Yann Gaston Mathé[1], Nicolas Do Huu[1], and Quentin Perron[1*]

[1] *Iktos, 65 rue de Prony, 75017, Paris, France*

E-mail: quentin.perron@iktos.com

1. Supporting Information available

All code and data can be found at *https://github.com/iktos/generation-under-synthetic-constraint*.

Spaya algorithm

The scheme in fig. S1 provides an overview of the retrosynthesis technology used in Spaya, and hence in the computation of the RScore. The process can be split in 2 blocks : the databases and the algorithm.

Databases

For this work, the chemical reaction database used was PISTACHIO,[1] which is developed by Next Move Software. This database provided approximately 85,000 chemical templates

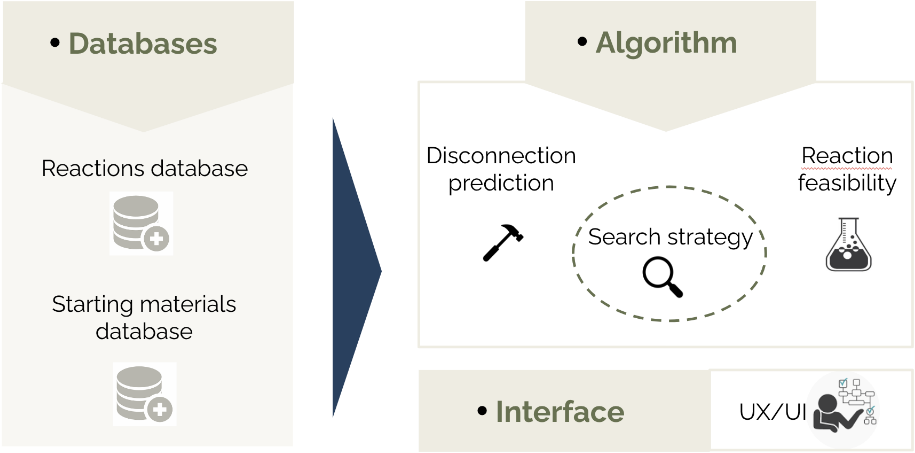

Figure S1: Overview of the retrosynthesis technology behind Spaya

and was used to train the single-step retrosynthesis model described below.

Spaya leverages starting materials from 17 different providers, which are detailed in table S1. These building blocks are then used as starting materials of synthetic routes.

Table S1: List of building blocks providers used by Spaya.ai

Provider	URL
1PlusChem	https://www.1pchem.com/
Advanced ChemBlocks	https://www.achemblock.com/
Appolo Scientific	https://www.apolloscientific.co.uk/
ChembBridge	https://chembridge.com/
Chem Div	https://www.chemdiv.com/
Chem Space	https://chem-space.com/
Chemtellect	https://chemtellect.com/
eMolecules	https://www.emolecules.com/
Key Organics	https://www.keyorganics.net/
Liverpool ChiroChem	https://www.liverpoolchirochem.com/
Life Chemical	https://lifechemicals.com/
molport	https://www.molport.com/
Mcule	https://mcule.com/
Mcule Ultimate	https://ultimate.mcule.com/
SpiroChem	https://spirochem.com/
TCI chemicals	https://www.tcichemicals.com/
Thermo Fisher	https://www.thermofisher.com/

Algorithm

The algorithm is comprised of three main components: a disconnection predictor, a search strategy, and reaction feasibility models. Two main approaches exist for the disconnection predictor: template-based or template-free models. In the template-based approach,[2] a model attempts to predict a reaction template before applying it to the input molecule. In the template-free approach,[3,4] the reactants are predicted directly from the input molecule. Spaya uses a the template-based approach, and the model is a feed-forward neural network that outputs a probability for each template. The training set consists of approximately 3 million reactions, with an associated retrosynthesis template that matches each reaction.

The search strategy used is a Monte-Carlo-Tree-Search (MCTS) algorithm.[5] When applied to automatic retrosynthetic algorithms, a node of a tree represents a set of molecules, and inheritance signifies that in the child node, one molecule was split into one or two molecules after the application of a retrosynthesis template.[6]

To improve the quality of the predicted reactions, Spaya incorporates proprietary reaction feasibility models. These models compare the predicted reactions to reference reactions from the literature in order to penalize predicted reactions with regioselectivity and chemoselectivity issues.

Impact of timeout on the RScore

To assess the impact of the timeout in the retrosynthesis process on the RScore, we run SpayaAPI on 1000 molecules sampled randomly from ChEMBL 24,[7] successively with 3 different timeouts: 1 minute, 3 minutes and 5 minutes.

The two boxplots on fig. S2a and fig. S2b show the positive gap when increasing the timeout respectively from 1 to 3 minutes, and from 3 to 5 minutes. On average, increasing the timeout from 1 minute to 3 minutes leads to an increase of 0.1 in the score. The first plot shows that the impact is the greatest for molecules which are unsolved with the RScore1mn, that in some cases obtain a high score with 3 minutes timeout. When increasing from 3

minutes to 5 minutes the timeout, the average increase in RScore is much lower at 0.03. There are still few unsolved molecules in 3 minutes that are solved with good a score in 5 minutes, but those data points can be considered as outliers. The two plots also show the intrinsic stochasticity of the algorithm: sometimes increasing the timeout leads to a decrease in the score, it corresponds to the box or dots that are below 0. The MCTS is a stochastic algorithm, hence the final score assigned to a molecule is intrinsically stochastic.

Generated molecules and optimization graph

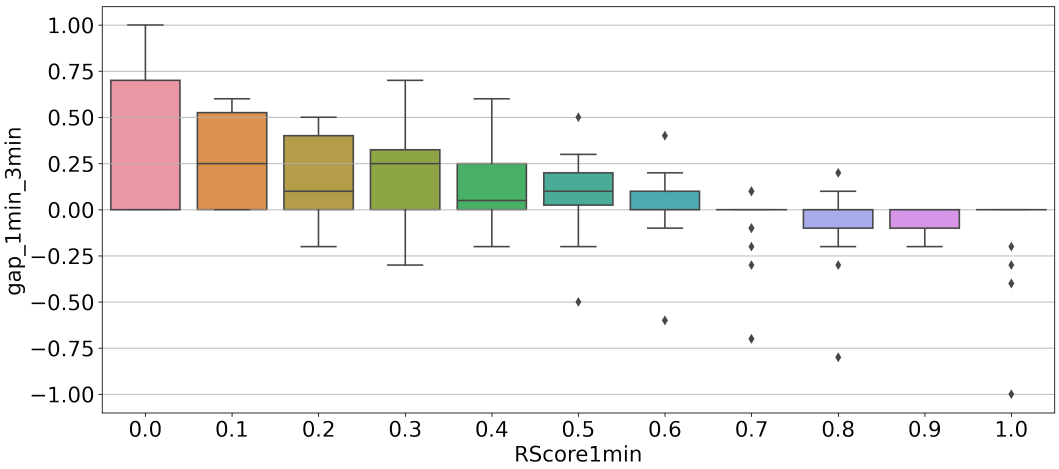

(a) Gap between RScore3min and RScore1min. The y-axis represesents the distribution of Rscore3min - RScore1min for a precise RScore1min.

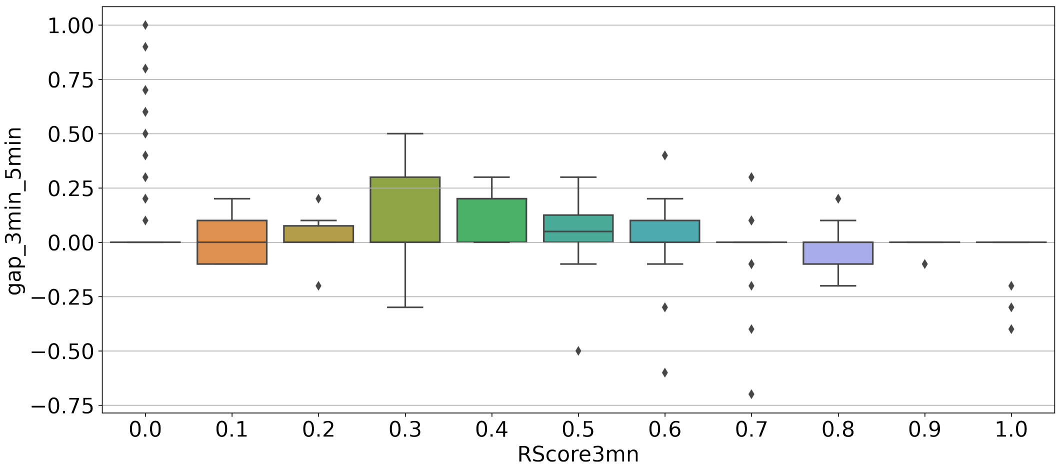

(b) Gap between RScore5min and RScore3min. The y-axis represesents the distribution of Rscore5min - RScore3min for a precise RScore3min

Figure S2: Impact of the timeout on the RScore, for 1000 molecules sampled from ChEMBL24.

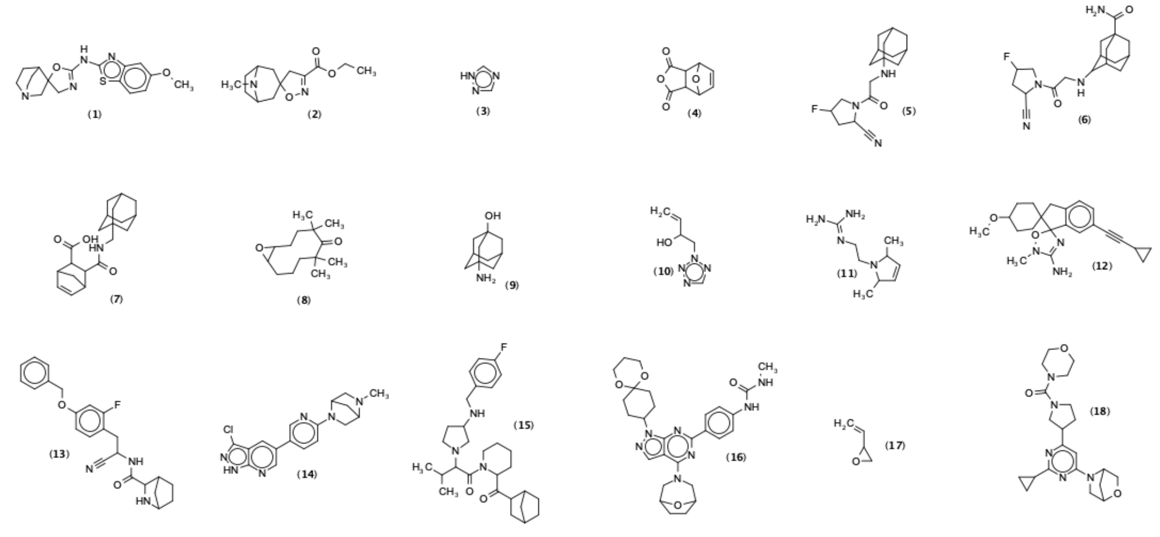

(a) Molecules around Chembl dataset.

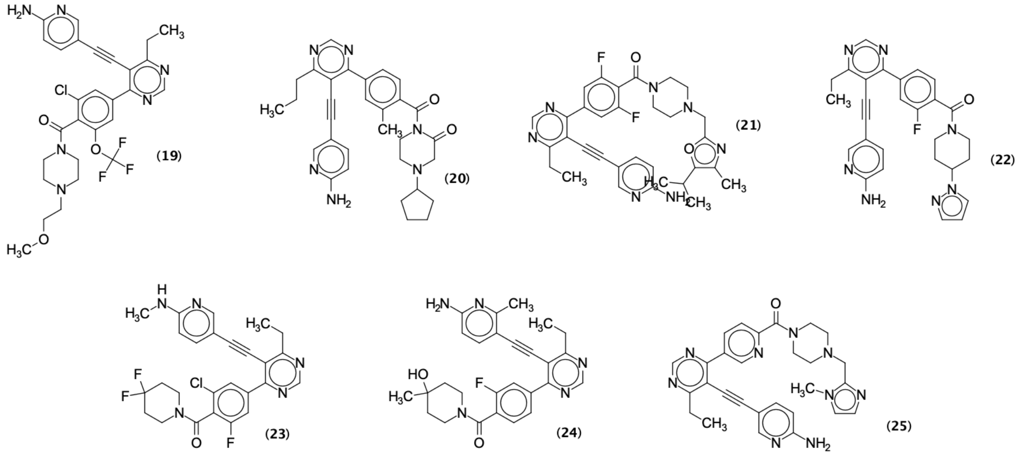

(b) Molecules around PI3K/mTOR dataset.

Figure S3: Example of molecules with a bad SA score (>3.5) but a good RScore (>0.4).

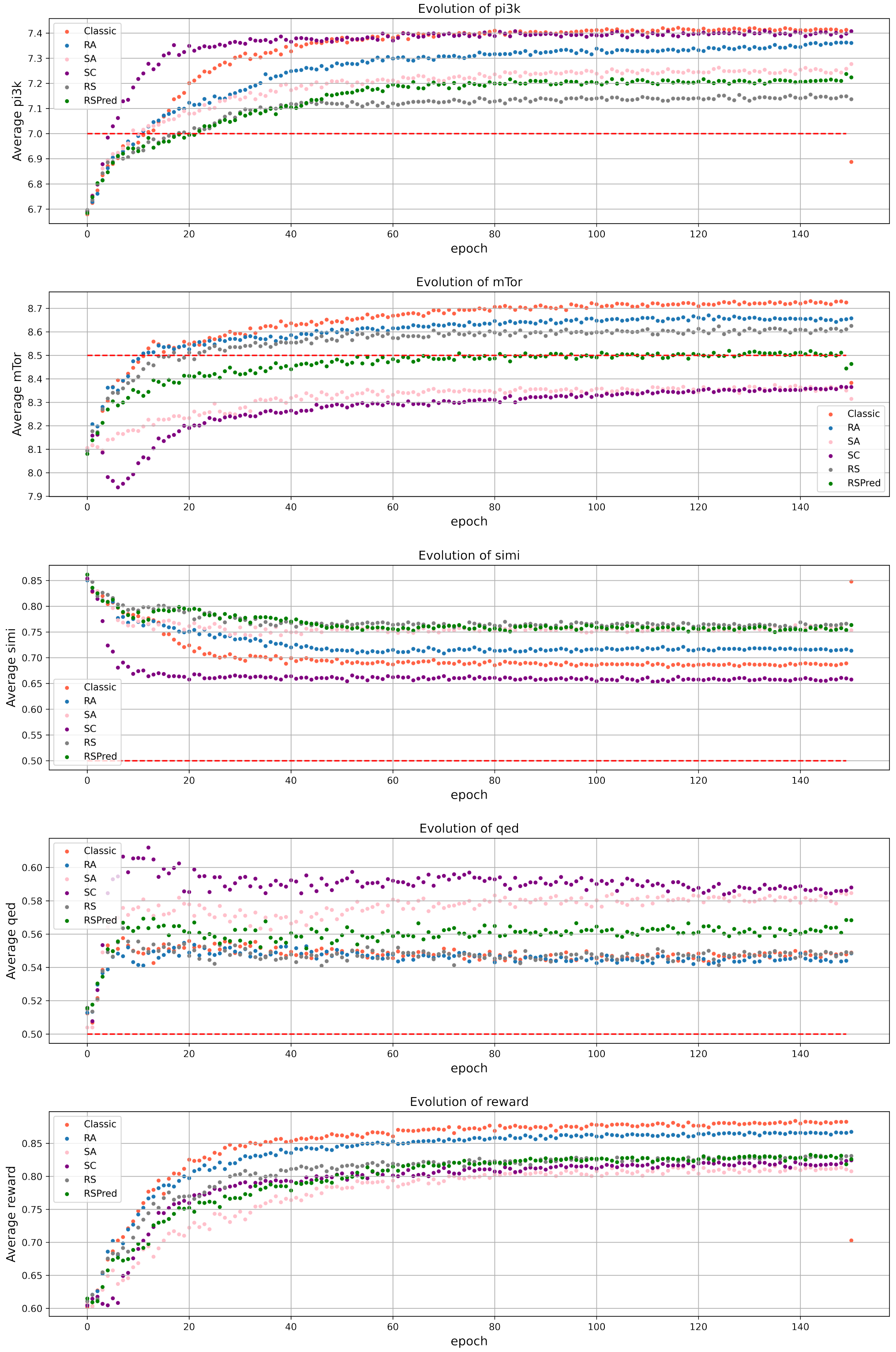

Figure S4: Evolution of 4 scoring functions : PI3K, mTOR, similarity, QED among epochs for 6 different generations around PI3K/mTOR dataset.

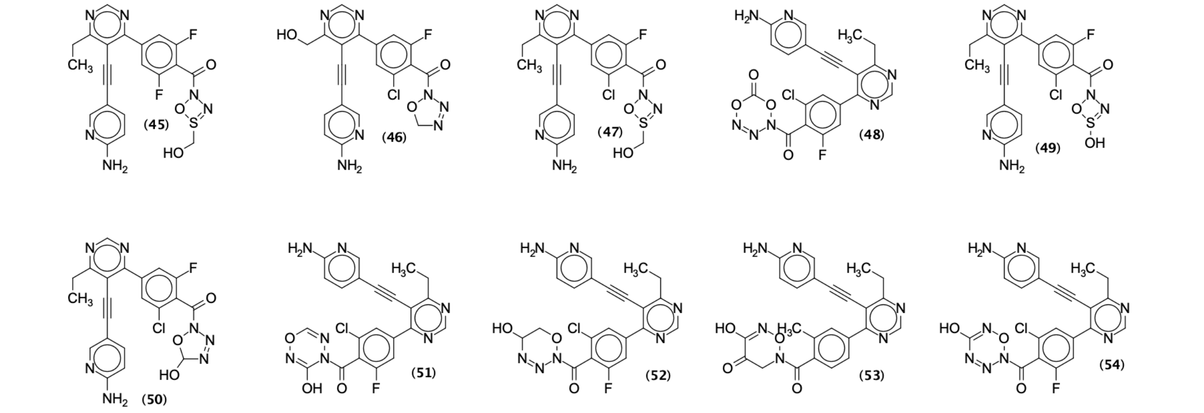

Figure S5: Top 10 molecules from PI3K/mTOR generation without any synthetic constraint.

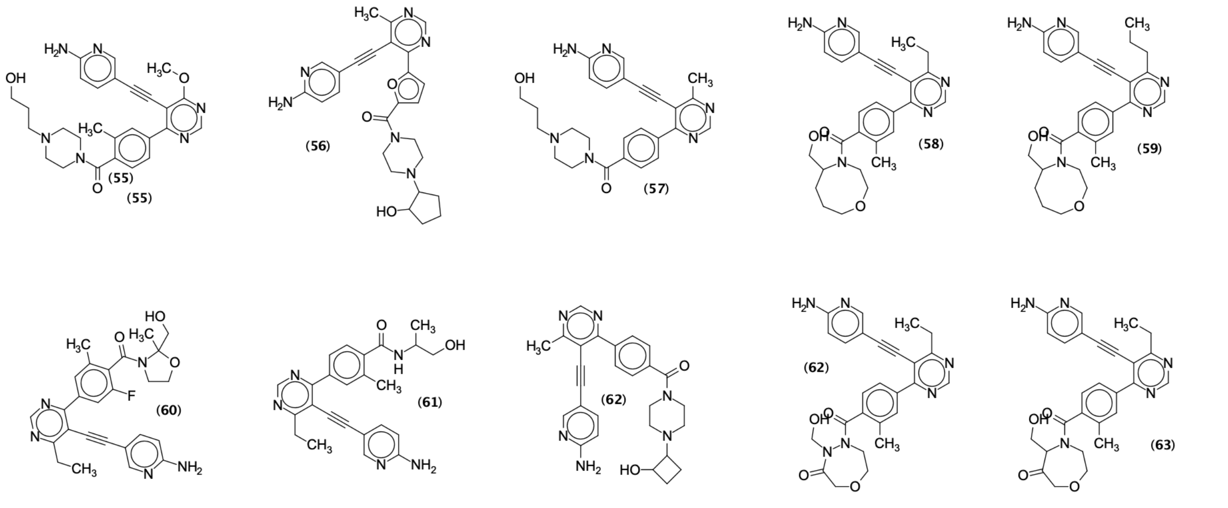

Figure S6: Top 10 molecules from PI3K/mTOR RA score constrained generation.

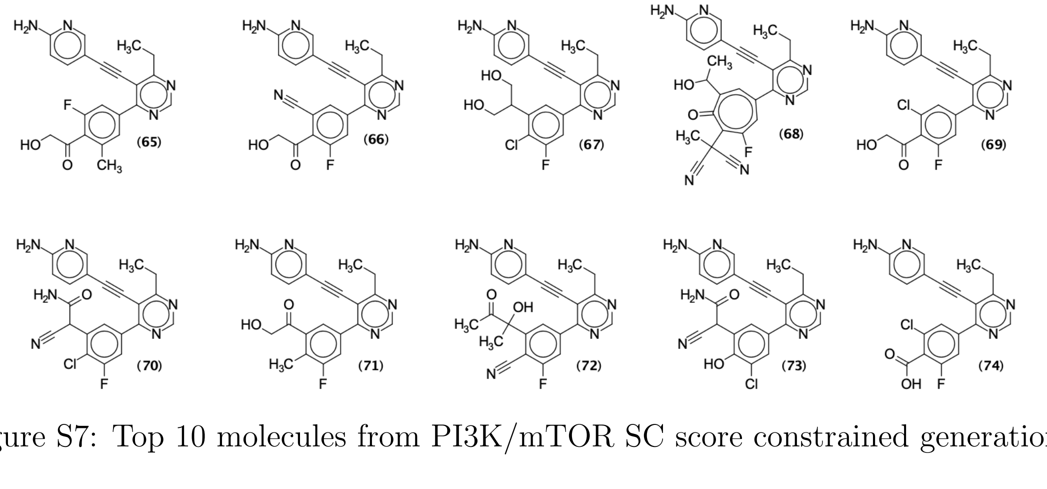

Figure S7: Top 10 molecules from PI3K/mTOR SC score constrained generation.

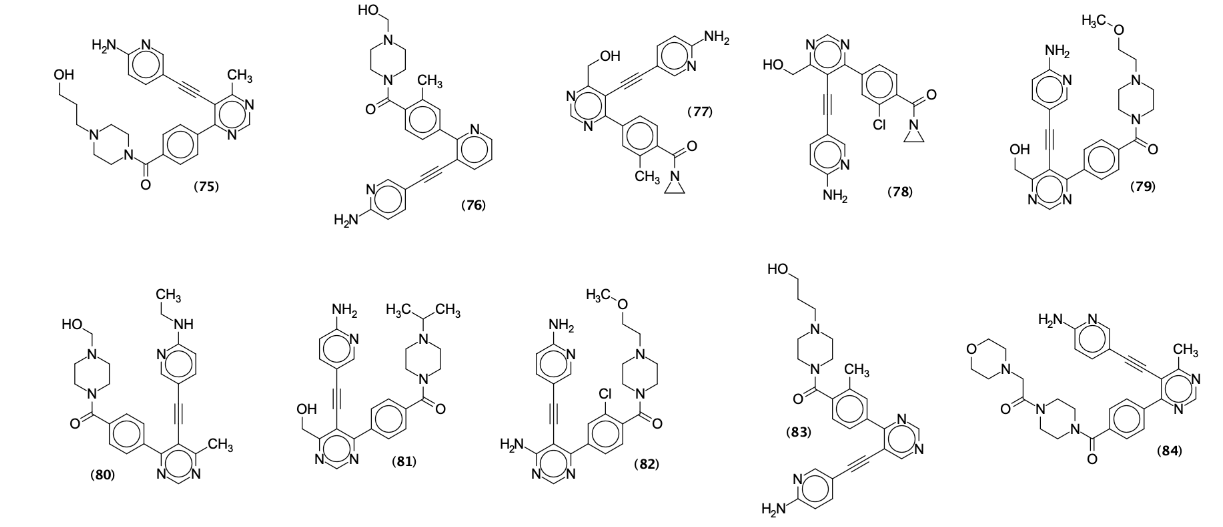

Figure S8: Top 10 molecules from PI3K/mTOR SA score constrained generation.

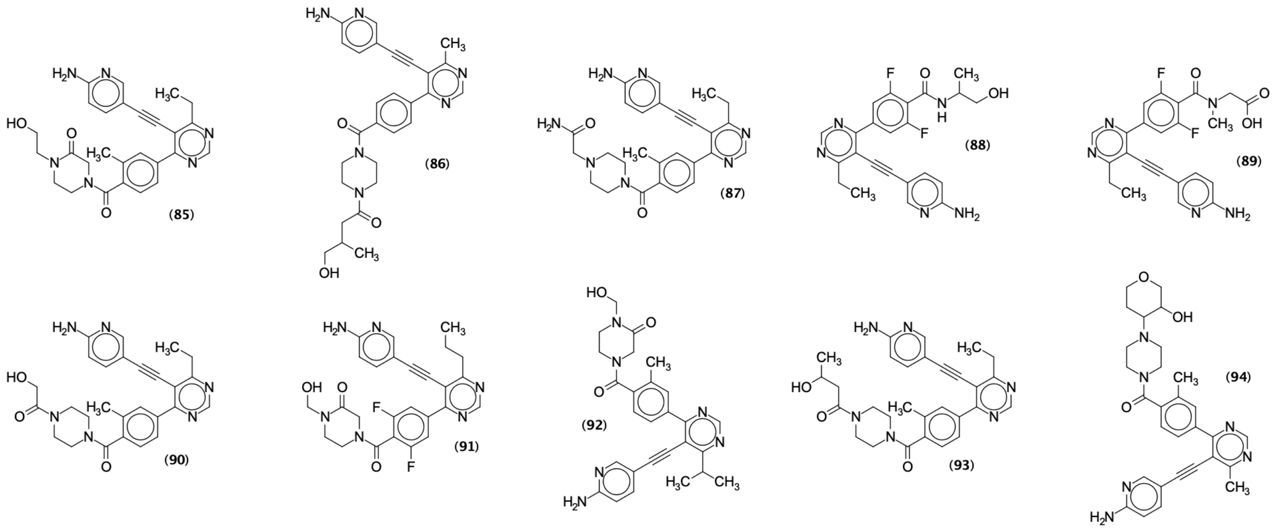

Figure S9: Top 10 molecules from PI3K/mTOR RScore constrained generation.

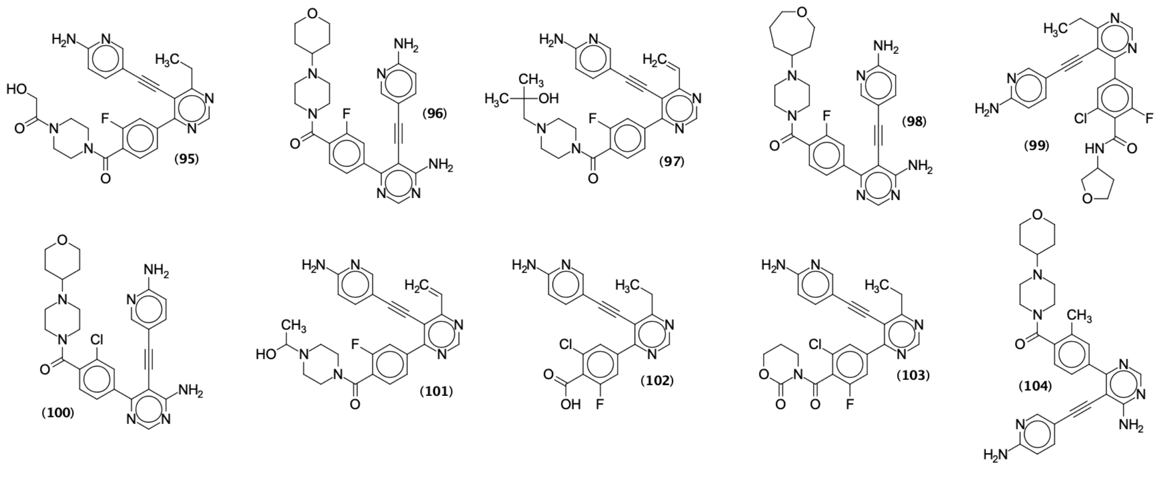

Figure S10: Top 10 molecules from PI3K/mTOR RSPred constrained generation.

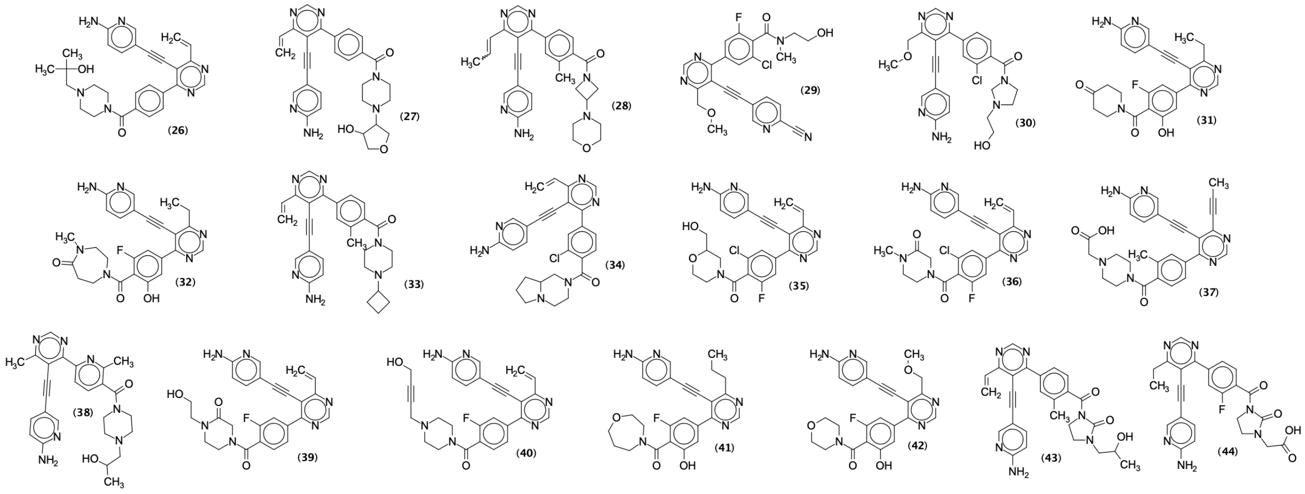

Figure S11: Molecules generated during RScore constrained generation.

References

(1) Software, N. M. PISTACHIO. `https://www.nextmovesoftware.com/pistachio.html/`, 2023.

(2) Segler, M.; Waller, M. Neural-Symbolic Machine Learning for Retrosynthesis and Reaction Prediction. *Chemistry (Weinheim an der Bergstrasse, Germany)* **2017**, *23*.

(3) Schwaller, P.; Laino, T.; Gaudin, T.; Bolgar, P.; Hunter, C.; Bekas, C.; Lee, A. Molecular Transformer: A Model for Uncertainty-Calibrated Chemical Reaction Prediction. *ACS Central Science* **2019**, *5*.

(4) Somnath, V. R.; Bunne, C.; Coley, C.; Krause, A.; Barzilay, R. Learning Graph Models for Retrosynthesis Prediction. **2021**,

(5) Browne, C. B.; Powley, E.; Whitehouse, D.; Lucas, S. M.; Cowling, P. I.; Rohlfshagen, P.; Tavener, S.; Perez, D.; Samothrakis, S.; Colton, S. A Survey of Monte Carlo Tree Search Methods. *IEEE Transactions on Computational Intelligence and AI in Games* **2012**, *4*, 1–43.

(6) Segler, M.; Preuss, M.; Waller, M. Planning chemical syntheses with deep neural networks and symbolic AI. *Nature* **2018**, *555*, 604–610.

(7) Mendez, D. et al. ChEMBL: towards direct deposition of bioassay data. *Nucleic acids research* **2018**, *47*.
